# Randomized Controlled Trials of Acupuncture (1997–2007): An Assessment of Reporting Quality with a CONSORT- and STRICTA-Based Instrument

**DOI:** 10.1155/2011/183910

**Published:** 2010-10-03

**Authors:** Richard Hammerschlag, Ryan Milley, Agatha Colbert, Jeffrey Weih, Beth Yohalem-Ilsley, Scott Mist, Mikel Aickin

**Affiliations:** ^1^Research Department, Oregon College of Oriental Medicine, Portland, OR 97216-2859, USA; ^2^Helfgott Research Institute, National College of Natural Medicine, Portland, OR 97201-4848, USA; ^3^Department of Physical Medicine & Rehabilitation, Kaiser Permanente Northwest, Portland, OR 97227-1110, USA; ^4^Fibromyalgia Research Unit, Oregon Health Science University, Portland, OR 97239-3098, USA; ^5^Program in Integrative Medicine and Department of Family & Community Medicine, College of Medicine, University of Arizona, Tucson, AZ 85724-5052, USA

## Abstract

The present study describes the development of a comprehensive quality of reporting assessment tool and its application to acupuncture RCTs from 1997–2007. This Oregon CONSORT STRICTA Instrument (OCSI) is based on the revised CONSORT guidelines as modified by the STRICTA recommendations for acupuncture trials. Each of the resulting 27 OCSI items were applied to English language prospective RCTs that compared acupuncture, using manual and/or electro-stimulation, to no treatment, a sham procedure, or usual biomedical care. The 333 RCTs that met inclusion criteria were dispersed among 27 countries and 141 journals. Mean quality of reporting score for all articles was 63.0% (SD 16.5). Mean OCSI scores revealed a 30.9% improvement over the ten-year period (*P* < .001). Our findings suggest that to enhance quality of reporting, authors should better attend to seven specific OCSI items in three categories: practitioner training, adverse events, and aspects of randomization and blinding (*n* = 5). The broad diversity in geographical origin, publication site and quality of reporting, viewed in light of the considerable room for improvement in mean OCSI scores, emphasizes the importance of making STRICTA as well as CONSORT more widely known to journals and to the acupuncture research community.

## 1. Introduction

Systematic reviews represent a retrospective, criteria-based approach for summarizing research findings [1–3]. By applying predetermined standards to identify the trials to be reviewed, and uniform criteria to evaluate the selected trials, bias in the quality assessment process is minimized. With the evidence based perspective being increasingly applied to complementary and alternative medicine (CAM) in the past decade, a wide variety of condition-focused systematic reviews have evaluated randomized controlled trials (RCTs) of acupuncture. MEDLINE alone lists over 170 of such reviews through 2009, many of which have, in turn, been summarized and analyzed [4–10]. As noted in these overviews, systematic reviews of acupuncture have employed a heterogeneous group of quality assessment instruments, that vary from the 5-item Jadad scale [11] and a modified 6-item Jadad scale [12–14], to the Cochrane Collaboration guidelines [15] and a range of broader scales containing up to 27 items [16].

In the present paper, we describe the development and application of a comprehensive quality of reporting instrument for rating RCTs of acupuncture, based on the revised CONSORT guidelines for RCTs [17] as modified by the STRICTA recommendations for acupuncture trials [18]. The Consolidated Standards of Reporting Trials (CONSORT) statement was created as a set of guidelines for use by journal editors, reviewers, and authors to increase the likelihood that RCTs submitted for publication would meet uniform standards for reporting [19]. The Standards for Reporting Interventions in Controlled Trials of Acupuncture (STRICTA) were crafted to modify *a single item* on the 22-item CONSORT list, referring to description of interventions. This item (CONSORT no. 4), calling for “precise details of the interventions for each group and how they were actually administered,” was considered too generic to be of value for improving reporting of acupuncture trials and was expanded to a 6-item list, with each item broken out into subitems, specifying the details of the acupuncture protocol to be reported [18]. Thus, STRICTA was created to complement, not substitute for, CONSORT.

It is important to recognize that the CONSORT statement is described as *“…a tool to improve quality of reporting of RCTs… (but not) …as a formal quality assessment instrument.” *[17, 20]. Despite this disclaimer, over 30 systematic reviews (predominantly focused on biomedical RCTs) have converted the CONSORT guidelines to a variety of scoring systems for evaluating quality of reporting. While several of these adaptations involved only a limited set of “essential” CONSORT items [21–24], many of the reviews devised a scoring system that utilized the full complement of CONSORT items [25–27], with some also breaking out selected items to create larger lists [28–32]. One review, published in Chinese, is the first to create a combined CONSORT and STRICTA quantitative assessment tool, applying it to the worldwide literature on RCTs of acupuncture for obesity [33].

A recent review sought to assess and compare the impact of CONSORT and STRICTA on the reporting quality in acupuncture trials during three time periods 1994-1995, 1999-2000, and 2004-2005 [34]. The review concluded that the reporting of selected CONSORT items has improved over time whereas no significant improvements were observed in STRICTA items. The authors also state that further exploration of the adherence to CONSORT and STRICTA within acupuncture RCTs is warranted, due to the limited number of studies sampled.

The present study describes the independent creation of a combined CONSORT- and STRICTA-based quality of reporting assessment tool, the Oregon CONSORT STRICTA Instrument (OCSI), and its application to acupuncture RCTs, across all conditions, published in the ten-year period following the October, 1997 NIH Consensus Development Conference on Acupuncture [35]. Our aims were to (1) develop a comprehensive quality of reporting assessment instrument based on two existing guidelines for clinical trial reporting, (2) examine mean scores per question, to inform the acupuncture research community as to which research design items are most often poorly reported or omitted, and (3) examine the overall scores per trial, to provide an indication of whether the quality of reporting in acupuncture RCTs has been improving over time. The development and application of OCSI have been presented in preliminary form [36].

## 2. Methods

### 2.1. Development of the Oregon CONSORT STRICTA Instrument (OCSI)

In creating the OCSI quality of reporting assessment tool, we followed the STRICTA recommendations of substituting 6 items, relevant to acupuncture and control group interventions, for item no. 4 of the 22 CONSORT items. Each of the resulting 27-item combined CONSORT and STRICTA guidelines was then converted to a question, retaining the CONSORT item sequence and wherever possible preserving the original wording for each item (Table 1). When clarification was necessary, we modified sentence structure but strove to remain as close as possible to the original wording. Where more significant changes were considered helpful, they were added to a separate list of modifications for future consideration.

A more significant problem in converting evaluative guidelines to questions was that most of the CONSORT and STRICTA items contained several embedded subitems that, while related, required modification to separate, sub-questions. For example, CONSORT item no. 11, concerned with reporting of blinding, states:

“Whether or not participants, those administering the interventions, and those assessing the outcomes were blinded to group assignment. If done, how the success of blinding was evaluated.” [17]

This compound item was converted to a multipart OCSI question, asking:

“Is it stated whether (a) participants, (b) those administering the interventions, and (c) those assessing the outcomes were blinded to group assignment; and (d) was the success of blinding evaluated?”

Each of the 27 OCSI questions was constructed to be scored *yes* = 2, *partial *= 1, *no* = 0, or *Not Applicable* = N/A, based on the composite scoring of its sub-questions. A question was scored *partial* if its sub-questions received a mix of ratings. If one or more sub-questions were scored *N/A*, the score for the question was based on the scores of the remaining sub-questions. *N/A* also could be assigned to an individual question; for example, OCSI question no. 7 regarding reporting of cointerventions was scored *N/A* if no adjunctive treatment (e.g., herbal medicine or moxibustion) was provided to the acupuncture group.

### 2.2. Article Selection Criteria

RCTs included for assessment met the following criteria: (1) publication date from November 1997 through October 2007; (2) prospective, randomized controlled trial; (3) human subjects; (4) English language; (5) full publication; (6) treatment with filiform acupuncture needles using manual and/or electrostimulation; (7) comparator/control group consisted of no treatment, a sham procedure, or usual biomedical care. Usual biomedical care was defined *a priori* as interventions usual and customary to biomedical conditions. These interventions were categorized as educational, behavioral, physical, or pharmaceutical. RCTs were excluded if acupuncture points were stimulated by means other than filiform needles, for example, ear tacks, intradermal needles, TENS, or laser.

Databases that were searched to identify articles included MEDLINE, the Cochrane Central Register of Controlled Trials, Alt HealthWatch, AMED, University of Maryland CAMPAIN, and the Oregon College of Oriental Medicine library database, which includes 16 non-MEDLINE journals of acupuncture and Oriental medicine. In addition, hand searches were performed of the reference lists from the WHO report on controlled clinical trials of acupuncture [37] and from 71 systematic reviews of acupuncture published between October 1997 and October 2007.

### 2.3. Application of OCSI

Seven individuals with experience in acupuncture research formed the OCSI group. Two members served as alternates, ensuring a group of 5 OCSI-raters over the course of the project. The group comprised a diverse range of backgrounds, all with experience in assessing RCTs of acupuncture, including three licensed acupuncturists, two nonpractitioners, a medical acupuncturist, and an acupuncture student. As a means of eliminating systematic bias, it was initially suggested to blind reviewers of RCTs to article information such as journal, author, and publication year [11]. An attempt to validate this recommendation was unsuccessful [38], and a recent assessment in acupuncture trials demonstrated limited difference in outcomes [34]. Based on these results, the OCSI Group was not blinded to article information.

From the RCTs that met the selection criteria, eight articles were initially chosen at random for scoring by the OCSI group (each of five raters) to assess face validity of the OCSI questions. Several group meetings were held to compare raters' scores per question on each of the eight articles and to seek consensus on scoring. The latter process included development of an OCSI manual that outlines criteria for scoring each question as *yes*, *partial*, *no*, or *N/A* (Appendix A).

Following the consensus process, the remaining articles were randomly distributed among five raters for individual scoring. Each article's score was divided by the total possible score (2 × [27 – *n* questions scored N/A]) and converted to percent. Scores for each OCSI question were entered into a database from which total OCSI scores per article were calculated. A second database contained extracted demographics from each article (e.g., country of origin, journal, year, condition). Results for the first eight articles were group consensus scores; results for the remaining articles were from single raters.

### 2.4. Statistics

For descriptive purposes, we computed and displayed averages of the individual question scores. For select questions, regression analysis was performed, and binominal 95% confidence intervals were computed for change of reporting over time. The distribution of all RCTs across OCSI score by decile is presented as a histogram.

Interrater agreement was assessed at a point in the study when low, middle, and high scoring articles could be discerned. Nine RCTs were randomly selected, three from each of these categories, in order to estimate agreement across the spectrum of quality. Conventional intraclass coefficients (ICCs) were computed from results with five reviewers each rating all nine articles.

Since China contributed the largest number of articles and had the lowest mean OCSI score by country, we plotted the mean OSCI scores by year for all countries, for China alone, and for all countries other than China, and fitted linear trends. Regression analysis was initially performed for all countries combined. A second regression analysis included a binary variable for China or all others as a covariate.

## 3. Results

### 3.1. Demographics of Included Trials

An initial search of the literature identified 410 RCTs that appeared to meet the inclusion criteria. Further review of the papers excluded 77 trials for reasons ranging from trials that were duplicate publications to trials that compared two forms of acupuncture. Inclusion criteria, including publication date from November 1997 through October 2007, were met by 333 acupuncture RCTs (Appendix B). These trials represented 27 countries with China accounting for the largest share (*n* = 78; 23.4%). Trials from the top five countries combined, including China, United States, Germany, Sweden, and United Kingdom, totaled 237 (71.2%) (Table 2). Asian countries other than China accounted for 26 (6.9%) trials. No attempt was made to determine how many RCTs were excluded on the basis of non-English language publication.

The 333 RCTs appeared in 141 journals, and the large majority of articles were listed in MEDLINE (*n* = 302; 90.7%). Of the total included trials, almost two-thirds (*n* = 209; 62.7%) appeared in biomedical journals, with the remainder (*n* = 124) in journals of complementary and alternative medicine.

### 3.2. OCSI Manual and Interrater Agreement

The initial face validity and consensus-building exercise led to clarification of OCSI items and drafting of a manual to inform scoring decisions. In most cases, the “Example and Explanation” section of the CONSORT document [17] and the similar section from STRICTA [18] were sufficient to facilitate its development. The manual proved especially helpful to set parameters for deciding between a score of *partial* versus *yes*, for example, item no. 22(c): “Presentation of *P* values alone will score *partial*; for full credit, authors must state precision as a confidence interval (CI).” The OCSI manual is presented as Appendix A.

Analysis of the group scoring (five members of OCSI group) of nine randomly selected articles demonstrates a high reliability (ICC) among raters (*r* = 0.99) as well as agreement within triads, low (*r* = 0.77), medium (*r* = 0.97), and high (*r* = 0.91) scoring ranges.

### 3.3. OCSI Scores per Question and per Article

**Figure 1:**
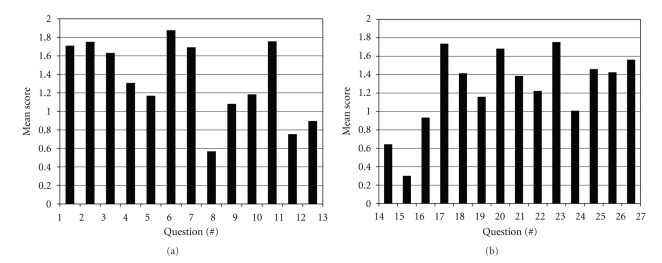
Distribution of scores of individual OCSI questions: (a) items 1–13; (b) items 14–27.

OCSI scores per individual question across all trials are presented in Figure 1. Since a rating of *partial* is scored as a 1, it is of interest that 7 of the 27 questions showed mean scores <1.0 (ratings between *no* and *partial*). Of these seven, only one was of STRICTA origin, question 8, asking for information on practitioner training, experience, and expertise. The other low-scoring questions are those that ask about reporting of sample size calculation (no. 12), randomization generation (no. 13), randomization implementation (no. 14), person responsible for randomization (no. 15), blinding (no. 16), and adverse events (no. 24). Of these poorly reported questions, 4 of 7 demonstrate trends of improvement over time, 3 of which reached statistical significance. These are questions: 12 (3.5% improvement *P* < .052; 95% CI = 0.000–0.071); 13 (4.9% *P* < .007; 95% CI = 0.014–0.084); 14 (6.2% *P* < .001; 95% CI = 0.028–0.094); 24 (4.6% *P* < .016; 95% CI = 0.009–0.083).

In contrast, 9 questions showed means ≥1.5 (of a possible 2.0) (Figures 1(a) and 1(b)). The highest scorers among these (in order of ranking) were questions asking for reporting of number and frequency of treatments (no. 6), primary and secondary outcomes (no. 11), scientific background, and rationale (no. 2), statistical methods (no. 17) and randomization included in title or abstract (no. 1).

**Figure 2:**
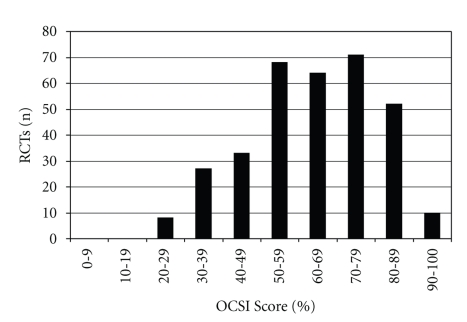
Distribution of OCSI scores across articles: scores binned by decile.

Distribution of OCSI scores per RCT is presented in Figure 2. The mean percent score for all articles was 63.0 (SD 16.5). The numbers of trials with OCSI scores higher than arbitrary values of 60, 70, and 80% were 197 (59.2%), 133 (39.9%), and 62 (18.6%), respectively. Mean score of RCTs from CAM journals (51.3% SD ± 15.1) was significantly lower than the mean from biomedical journals (69.9% ± 13.1) (*P* < .001; 95% CI = 15.4–21.8%). Of the five countries publishing the greatest number of RCTs (China, USA, Germany, Sweden, and UK), the mean OCSI score of articles from China (45.2% ± 14%) was significantly lower (*P* < .001; 95% CI = 21.0–28.2%) than mean scores of articles from the other four countries (69.8% ± 2.7). The top five journals publishing trials that met our inclusion criteria are *J Tradit Chin Med *(*n* = 39)*, Internat J Clin Acupunct* (*n* = 14), *Pain* (*n* = 13), *J Altern Complement Med* (*n* = 12), and *Acupunct Med* (*n* = 11). Of these five journals, *The Journal of Traditional Chinese Medicine, *which accounted for the largest percentage of publications (11.7%), demonstrated the lowest mean OCSI score for its acupuncture RCTs (39.6%).

**Figure 3:**
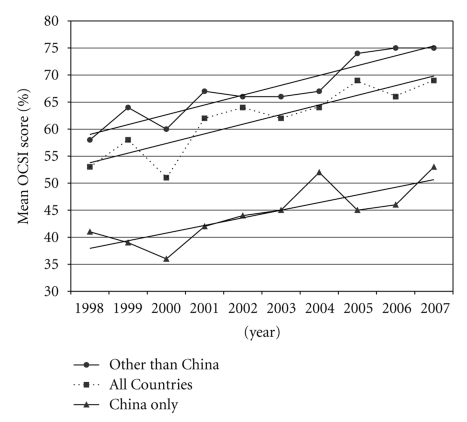
OCSI scores over time grouped by RCTs from all countries, China only, and all countries other than China.

Figure 3 shows mean OSCI and modeled scores over time for trials from all countries, from China alone, and from countries other than China. The mean OCSI scores from all countries demonstrate a significant improvement of 30.9% over the ten-year period (*P* < .001). Significant improvements are found in RCTs from China 37.3% (*P* < .005) and countries other than China 32.2% (*P* < .005) over time. The modeled mean 1998 OCSI score of RCTs from China (36.1%) is significantly lower (*P* < .01) than the corresponding score from countries other than China (59.6%). The improvement on OCSI scores from China (1.4% per year, *P* < .005) as compared to the improvement in all other countries (1.7% per year, *P* < .001) is not significantly different (*P* < .42).

## 4. Discussion

Creation of OCSI required two main steps: conversion of the combined guidelines into questions, including breakout of each multicomponent item into a nested set of sub-questions, and development of a manual that outlines criteria for scoring each question as *yes*, *partial*, *no*, or *N/A*. The former step resulted in a lengthier scoring instrument than we had initially envisioned (63 sub-questions grouped into 27 main questions). The latter, based in large part on the detailed rationales presented with CONSORT [17] and STRICTA, proved essential for applying OCSI and achieving interrater agreement.

The acupuncture RCTs identified for scoring by OCSI revealed a strikingly broad diversity in both geographical origin and publication site, a finding supported by the recent review by Prady et al. [34]. These demographics, viewed in light of the considerable room for improvement in mean percentage OCSI scores of acupuncture trials appearing in both CAM (51.3, ±15.1) and biomedical (69.9, ±13.1) journals, emphasize the importance of making CONSORT and STRICTA more widely known and applied. This point is highlighted by a recent survey of high impact journals (*n* = 165), of which only 62 (38%) mentioned the CONSORT statement in their online “Instructions to Authors” while 23 (14%) stated that a completed CONSORT statement was a condition of submission [39]. Lack of adequate reporting is not unique to English language publications. A recent review of adherence to CONSORT in 142 RCTs from five leading Chinese medical journals indicated an overall low quality of reporting [40–42]. The translations of both CONSORT and STRICTA into several Asian languages should help the dissemination effort [43–46].

In regard to the seven lowest-scoring OCSI questions (those with mean score <1.0), several recommendations can be made to the acupuncture research community. The sole question from STRICTA within this group (question no. 8) is important since practitioner training and experience may significantly affect the outcome of a trial. The low reporting of this item is consistent with the recent assessment of STRICTA items [34] and is also reflected in the low ranking of the utility of this question by authors of acupuncture trials [47]. The importance of reporting practitioner training and experience is clearly emphasized in the revised STRICTA Recommendations [48]. Five items from this group (questions nos. 12–16) pertain to aspects of the randomization and blinding procedures. As recommended in CONSORT, transparent reporting of these items is necessary to ascertain that selection and performance biases have been reduced [17]. A further low-scoring question (no. 23) is that calling for reporting of adverse events. Data on this topic is of general importance and is a particularly relevant comparator in RCTs of acupuncture versus biomedical standard of care [49].

The mean OCSI score (63.0%; median 64%) of the 333 acupuncture RCTs included in the present review indicates a moderate level of adherence to the CONSORT and STRICTA guidelines. This score is somewhat higher than those from a study that assessed observance of STRICTA as well as a selected set of CONSORT recommendations in samplings of acupuncture RCTs appearing both before and after publication of each set of guidelines [34]. It is also of interest that our subset of 209 acupuncture RCTs that appeared in biomedical (in contrast to CAM) journals received a mean score of 69.9%, similar to the adherence levels to CONSORT in assessments of RCTs of psychotropic pharmaceuticals [28], general medicine interventions [50, 51], endocrinology [23], and surgical procedures [27, 52]. 

Although a significant trend toward an annual increase in reporting quality is apparent in our findings, the 68.7% mean OCSI score for the first three-quarters of 2007 (the most recent period evaluated) indicates a continuing need to improve reporting in acupuncture RCTs. As a secondary analysis, composite scores were examined of both the CONSORT- and STRICTA-based questions. Analysis of CONSORT-based questions reveal an improvement of 18% over the 10-year period (*P* < .001, 95% CI = 0.011–0.025), with STRICTA-based questions improving by 17% (*P* < .001; 95% CI = 0.006–0.017). While a similar increase in CONSORT-related reporting in acupuncture RCTs was observed by Prady et al. [34], the STRICTA-related increase was not previously observed, a difference likely related to the inclusive (present study) rather than sampling (Prady et al.) approaches utilized. Although the increased trends are apparent, the overall mean OCSI score (63%) implies that reporting of the acupuncture intervention (STRICTA) needs improvement as much as reporting of general aspects of research design (CONSORT).

Interpretation of our results is impacted by the included RCTs from China, which comprise, when ranked by country, both the largest number of trials and the lowest mean OCSI score (Table 2). When RCTs from China are excluded, the mean OCSI score increases from 63.0 (16.5) to 68.4 (13.5). A matter of concern is that many RCTs from China either did not actually randomize participants or utilized inadequate procedures [53]. It is likely that this finding adversely impacted, the OCSI score of RCTs from China. Although, over the decade examined the OCSI scores from China are consistently lower than those form all other countries, the translations of both CONSORT and STRICTA into Chinese should help to close the gap in quality of reporting [44]. A recent national effort in China to improve trial design of acupuncture RCTs should also contribute to enhanced reporting quality [54].

Two further cautionary notes apply when considering interpretations of OCSI scores. First is the somewhat surprising finding that 54% of authors responding to a survey on their use of STRICTA reported that some checklist items were removed in the journal review process in response to editorial concern regarding space constraints [47]. If this is indeed a widespread occurrence, journals should be encouraged to request alternate means for authors to provide STRICTA and CONSORT details, for example, electronic links to an expanded methods section. Second, in calling for acupuncture RCTs to meet reporting standards set by CONSORT and STRICTA, it should be considered that many acupuncture trials have been and continue to be designed as “early phase” research [55]. This does not mean that such trials should be allowed a lower standard of reporting quality but it does indicate that the standards should be applied in a manner appropriate to the early stage of the research, when issues concerning patient recruitment, compliance, and retention are as critical to assess as are “hint of efficacy” and maintenance of benefit. For example, an early phase study should not “lose credit” for lack of reporting a sample size calculation if, in fact, no preliminary data existed on which to base such a calculation. Accordingly, in this case, the OCSI group decided that full credit should be awarded if a rationale was provided for the omission of a sample size calculation.

In addition to the limitations of our findings discussed above, there are several areas to consider for improving the OCSI instrument itself. For example, our decision to keep the numbering, wording, and grouping of the questions similar to those of the parent guidelines should be reconsidered since it may be of advantage to split out some of the sub-questions into stand-alone questions. One example would be to track, as a separate full question, whether the success of blinding study participants was evaluated. In the current OCSI format, evaluation of blinding is scored as a sub-question grouped with related sub-questions that ask whether study participants, practitioners, and assessors were reported as blinded (question no. 16). While this grouping is appropriate as a single CONSORT *guideline,* a greater impact on overall scoring might be achieved if this sub-item was a separate question.

One can also argue that the comprehensiveness of OCSI is unnecessary to identify flaws in reporting and we should seek instead to identify a core set of questions that produce a quality score sufficiently similar to the total OCSI score. A potentially fruitful approach would be to survey stakeholders in the quality of reporting arena for sake of creating a weighting system for questions. Such an approach would minimize the “fatal-flaw” scenario of the unweighted instrument wherein a high score is achieved despite failure to report an item generally regarded as essential to “good reporting.” For this initial creation and application of OCSI, however, our goal was not to identify those items most essential for rigorous research design but to provide a means to assess comprehensive reporting in acupuncture RCTs, consistent with the aims of CONSORT and STRICTA.

Finally, in regard to application of the present findings, it is important to stress that quality of reporting is only a first step toward assessing the evidence base. For example, receiving high scores for reporting interventions may be of questionable clinical relevance if what is being reported are inadequate or inappropriate treatments that, in turn, may have contributed to reduced efficacy. Similarly, participant selection criteria, outcome measures, or statistical procedures that are clearly reported may score high on OCSI despite being insufficient or inappropriate matches to the research question, the condition studied, the intervention provided, or the analysis of results. Thus, while OCSI may be regarded as a comprehensive tool for assessing quality of reporting, additional instruments as well as expert practitioner and statistician panels are needed to assess adequacy of the intervention and clinical relevance of the overall research design [56–60]. To this end, our aim is to improve OCSI within the context of a more complete strategy for assessing and building an evidence base for acupuncture. We also encourage the use of OCSI as a model for developing quality of reporting tools for other therapies that have already created official and unofficial CONSORT extensions similar to STRICTA, such as herbal interventions [61] and homeopathy [62].

## Figures and Tables

**Table 1:** Oregon CONSORT STRICTA instrument (OCSI).

Item number	Paper section	Question
1	Abstract	Is there an explicit statement that patients were randomly assigned to interventions?

2	Introduction/Background	(a) Is scientific background provided and (b) is the rationale explained?

3	Methods	(a) Are the eligibility criteria (inclusion and exclusion criteria) stated and (b) are the setting(s) and location(s) where the data was collected described?

4	Methods	(a) Is the style of acupuncture stated? (b) Is the rationale presented for the selection of acupuncture points? (c) Was the rationale justified?

5	Methods	Are the following parameters of needling presented? (a) Points used (uni/bilateral) (b) Number of needles inserted (c) Depth(s) of insertion (d) Response elicited (e.g., de qi) (e) Needle stimulation (manual or electrical) (f) Needle retention time (g) Needle type (Material and/or manufacturer, gauge, and length)

6	Methods	Are the (a) number and (b) frequency of treatments stated?

7	Methods	Are details of the acupuncture group cointervention(s) presented? (e.g., moxa, cupping, life-style advice, plum-blossom needling, Chinese herbs)

8	Methods	Are descriptions provided of the (a) duration of practitioner training, (b) length of clinical experience, and (c) expertise in specific condition?

9	Methods	(a) Is the intended effect of the control or comparison intervention presented? (b) Were the specific explanations given to patients of the treatment and control interventions presented? (c) Are details for the control or comparison intervention presented? (d) Are sources provided, that justify the choice of the control or comparison intervention?

10	Methods	Are there statements of (a) specific objectives and (b) hypotheses to be tested?

11	Methods	(a) Are primary and (if applicable) secondary outcome measures clearly defined? (b) Are there statements (when applicable), regarding any methods used to enhance the quality of measurements, for example, multiple observers or training of assessors?

12	Methods	(a) Is there a statement regarding how the sample size was determined, and (b) if applicable, an explanation of any interim analyses and stopping rules?

13	Methods	(a) Is the method presented that was used to generate the random allocation sequence, and (b) if applicable, details of any restriction (e.g., blocking, stratification)?

14	Methods	(a) Is the method presented that was used to implement the random allocation sequence, (b) with clarification as to whether the sequence was concealed until interventions were assigned?

15	Methods	Are there statements as to (a) who generated the allocation sequence, (b) who enrolled participants, and (c) who assigned participants to their groups?

16	Methods	Is it stated whether or not (a) participants, (b) those administering the interventions, and (c) those assessing the outcomes were blinded? and (d) was the success of participant blinding evaluated?

17	Methods	(a) Were the statistical methods stated that were used to compare groups for primary outcomes? (b) Were the statistical methods stated that were used for additional analyses such as subgroup or adjusted analyses?

18	Results	(a) Is the flow of participants through *each* stage quantitatively described, and (b) if protocol deviations are reported, were reasons presented?

19	Results	(a) Are dates provided that define the period of recruitment? (b) Is the length of followup (on-treatment and posttreatment) reported?

20	Results	(a) Are baseline demographics and (b) clinical characteristics presented for each group?

21	Results	(a) Is the number of participants in each group included in each analysis? (b) Was the “intention to treat” analysis presented? (c) When feasible, are the results stated in absolute numbers (e.g., 10 of 20, not just 50%)?

22	Results	For each primary and (if applicable) secondary outcome, is (a) a summary of results presented for each group, (b) the estimated effect size presented for each between-group difference (e.g., SD), and (c) the precision of the effect size presented for each between-group difference (e.g., confidence interval (CI))?

23	Results	If additional subgroup analyses and/or adjusted analyses are reported, is it stated whether they were prespecified or exploratory, that is, *not* prespecified?
24	Results	Are all important adverse events or side effects presented for each intervention group?

25	Discussion	Is an interpretation of the results presented that takes into account (a) study hypotheses, (b) sources of potential bias or imprecision, and (c) the potential dangers associated with multiple analyses and outcomes?

26	Discussion	Is the generalizability (external validity) of the trial findings discussed?

27	Discussion	Is a general interpretation of the results presented, in the context of current evidence?

Instructions. OCSI evaluates how well an “item” is reported, not whether it was appropriate or adequate. When scoring each question, consider the following. (i) If you were a reviewer of the paper, would you be satisfied with what is reported? (ii) If you were attempting to reproduce the findings, is there sufficient reporting of details to allow you to do so?

Note. Items 4–9 from STRICTA (MacPherson et al., 2002) substitute for item 4 of CONSORT (Altman et al., 2001) [17, 18].

**Table 2:** Distribution of OCSI scores by country.

Country	Articles (*n*)	Mean Score (%)
China	78	45.2
USA	58	69.7
Germany	45	73.2
Sweden	33	64.4
UK	23	72.5
Norway	14	68.7
Italy	11	59.6
Taiwan	11	58.8
Australia	10	72.3
Austria	7	61.6
Japan	7	62.8
Spain	4	85.4
Brazil	3	71.2
Croatia	3	46.7
Denmark	3	63.7
Hong Kong	3	85.0
Iran	3	61.4
Israel	3	59.3
South Korea	3	60.9
Canada	2	78.5
Switzerland	2	75.3
Turkey	2	62.7
France	1	80.0
Greece	1	48.0
Ireland	1	65.0
Singapore	1	72.0
Thailand	1	62.0

Total	333	63.0

## Appendices

### A. Oregon CONSORT-STRICTA Instrument (OCSI) Manual

#### A.1. General Instructions

- When scoring each question consider the following.
  - If you were a reviewer of the paper, would you be satisfied with what is reported?
  - If you were attempting to reproduce the findings, is there sufficient reporting of details to allow you to do so?
- When multiple components (e.g., (i), (ii), (iii)) or “and” statements are listed for a question, all aspects must be met to receive full credit.
- Except in regard to question no. 1, if the information is placed ANYWHERE in the document, the question can receive full credit.
- For questions 4–9, which refer to STRICTA, nos. 4–8 pertain ONLY to the acupuncture aspects of the study, while no. 9 pertains to all types of control or comparison groups.
- Practitioners are almost always NOT BLINDED in acupuncture research; hence no. 16(b) is usually scored “N/A” (see no. 16(b) for further details).

#### A.2. Instructions per Question

No. 1. It must state in the abstract or title that participants were allocated to interventions in a randomized manner, that is, “random allocation,” “randomized,” or “randomly assigned.”

No. 2(a). “Scientific background” pertains to (i) the previous research and (ii) the current understanding of the disease. No. 2(b). “Rationale” pertains to the justification for the trial and/or the expected effects/benefits of the treatment.

No. 3(a). The distinction between inclusion and exclusion criteria is unnecessary; for example, an exclusion criterion may be listed as a negative within the inclusion criteria. Stating the patient demographics and clinical characteristics after recruitment (e.g., post hoc demographics) *does not* qualify as eligibility criteria. No. 3(b). “Settings,” for example, private practice or outpatient clinic and “Locations,” for example, medical center or acupuncture college, can be implied from the author affiliation information.

No. 4(a). “Style of acupuncture” can be implied from the author affiliation information; for example, authors were from the Beijing TCM hospital, so style is TCM. If the protocol utilized a single acupoint or only lists the names of the acupoint(s), the style still needs to be stated. No. 4(b). For example, the acupoints were chosen on the basis of text books, practitioner survey, or prior research. No. 4(c). “Justified,” for a YES score, requires citing journals, texts, practitioner survey, or an expert panel.

No. 5(a). Unilateral or bilateral can be implied. No. 5(b). If the treatment protocol is individualized, then the authors must state the range of needles utilized (e.g., 8–14 needles). This may be implied from no. 5(a). No. 5(c). If the treatments were individualized, the depths must be given as a range. Depths can be expressed in *Cun*, mm, or anatomical depth (e.g., subcutaneous tissue or muscle). No. 5(d). “Response” refers to needle sensations such as, de qi (TCM), muscle twitch (trigger point), or muscle contraction (electroacupuncture). No. 5(e). “Stimulation” refers to techniques in manual and electroacupuncture, for manual acupuncture: lifting, thrusting, or rotating; for electroacupuncture: the current, frequency, and amplitude. No. 5(f). If the treatments were individualized “retention time” must be reported as a mean and range. No. 5(g). It must state the gauge, length, and material and/or the manufacturer of the needles.

No. 6(a). If treatments were individualized, then the mean and range of the number of treatments must be stated. No. 6(b). If the protocol was one treatment, then the frequency is “N/A”.

No. 7. It refers only to cointerventions within the scope of acupuncture licensure. If the protocol specifies the option of prescribed self-help treatments, for example, exercise advice, stretching, or qi gong, these must also be described.

No. 8(a). “Duration of practitioner training” can be implied, for example, LAc or a similar indication of formal training, (e.g., German Physicians Society of Acupuncture). No. 8(b). “Clinical experience” refers to number of years in practice as an acupuncturist. No. 8(c). “Expertise” must include experience, as an acupuncturist, with the condition under investigation.

Nos. 9(a–d). A no-treatment control is to be scored in the same manner as a sham or comparison control. No. 9(a). “Intended effect” refers to the specific rationale for choosing the control (including no treatment), in relation to the research question and methodology. No. 9(b). “Specific explanations” refers to the specific wording the patient receives regarding the treatment and control procedures. This information is needed, for example, since describing a sham procedure as “a type of acupuncture” may affect outcomes differently than stating “it isn't acupuncture.” No. 9(c). “Details” refers to a precise description of the control intervention including (if applicable, e.g., invasive sham) the parameters of needling (nos. 5(a–g)). This includes “no treatment” controls, for example, who may have been required to keep diaries. If no tracking was performed, it must be stated. No. 9(d). “Justification” refers to citing journals, textbooks, practitioner survey, or an expert consensus panel.

No. 10(a). If objectives are clearly implied but not “specifically stated,” a “Yes” may be scored. No. 10(b). The actual word “hypothesis” or a valid substitute, for example, theory, must be used.

No. 11(a). It must explicitly state (i) the primary outcome(s) *and* (ii) how it (they) was measured. If the primary outcome was not *specifically* indicated, partial credit can be awarded. (The primary outcome is usually used in the sample size calculation.) No. 11(b). If particular steps were taken to increase the reliability of the measurements, sufficient details of the steps must be presented; for example, range of motion was assessed with a goniometer, and three successive measurements were averaged to increase reliability.

No. 12(a). It must include statements regarding estimated effect size of the intervention based on (i) previous research and (ii) estimated attrition rate. If the study was a pilot then a statement such as “No data exists upon which to base a sample size calculation” must be included. No. 12(b). “Interim analysis and stopping rules” pertains to long-term trials and may not be applicable to trials of shorter duration. If authors report that interim analyses were performed, the number of assessments and statistical tests must be reported.

No. 13(a). Sufficient and specific information must be provided regarding the methods utilized to generate the random allocation scheme, for example, a *computer*-generated table. Simply stating that patients were randomly assigned is not sufficient to determine the likelihood of bias. No. 13(b). If randomization restrictions were utilized, for example, stratification or blocking, the methods and details must be stated.

No. 14(a). “Implementation” refers to how the random assignment was applied; for example, a *sealed envelope* containing the computer generated-group assignment was placed in the patient's chart. No. 14(b). Concealment is required to prevent foreknowledge of and introduction of bias in treatment assignment, for example, the sealed envelope with group assignment was opened after the patient passed the baseline neurological exam and received a TCM differential diagnosis.

No. 15(a). It must *specifically* state the person responsible for generating the randomization. No. 15(b). It refers to the *specific* person(s) responsible for consenting the participants. No. 15(c). It refers to the person who assigned participants to their respective randomized groups. This may likely refer to the person who informed practitioners, directly or indirectly, of the participant's group assignments. “Indirectly” may involve the preplacement of the group assignment in the patient chart. If “this” person also generated the randomization scheme, the authors should report how the potential for biased group assignments was addressed (e.g., randomization schedule was kept in a locked closet from “this” person).

No. 16(a). It must state that patients were blinded to group assignments, for example, a statement that the trial was single-blinded. If the trial was a comparison of active treatments, for example, acupuncture versus massage, this will be scored N/A. No. 16(b). This will usually be scored N/A. In the rare case that the study did blind the practitioners, there must be an explicit statement regarding the method utilized. No. 16(c). It must state whether or not the persons performing outcome measures were blinded to group assignments. This is more important with subjective outcomes, less so with objective outcomes where there is less chance or bias. No. 16(d). For full credit, authors must (i) state that the success of participant blinding was evaluated and (ii) present the results of the assessment. This is N/A if acupuncture was compared to another active treatment (e.g., standard care or massage).

No. 17(a). It must specifically state the statistical methods used to compare groups for primary outcomes. No. 17(b). If additional analyses were performed, the specific statistical methods used must be reported for full credit.

No. 18(a). A diagram is recommended but not necessary. For full credit, authors must state the number of participants who were (i) evaluated for potential enrollment, for example, excluded due to inclusion/exclusions criteria and declined enrollment (ii) randomized, (iii) received treatment, (iv) completed treatment, and (v) analyzed for the primary outcome. No. 18(b). “Protocol deviations” pertains to the nature of protocol deviations after randomization, for example, departures from the protocol including unplanned changes to the intervention, data collection, examinations, and methods of analysis, or the specific reasons for patient exclusions after randomization, including drop-outs.

No. 19(a). It must state the start and finish dates of the recruitment period, to place the study in historical context. No. 19(b). It must state the length of time for the followup period; for example, participants were followed for a total of 6 months, 3 months during treatment and 3 months of posttreatment followup.

No. 20(a). It must present the demographics, for example, age, gender, ethnicity. No. 20(b). It must present the clinical characteristics, for example, duration of condition, for each group.

No. 21(a). For full credit, it must state the “*n*” per each analysis, since the actual “*n*” may vary per outcome. If no dropouts occurred, he “*n*” can be implied. No. 21(b). Must use an “intention to treat” analysis (or a valid substitute, e.g., per protocol or on treatment analysis) to avoid bias associated with non-random loss of participants. If there were no dropouts then a “Yes” is scored. No. 21(c). It must state absolute numbers, for example, reduction of VAS pain from 10 to 5 or from 6 to 3 not just a 50% reduction.

No. 22(a). It must state the results or use a table/graph, for example, the mean and standard deviation of the measurements. No. 22(b). All results must indicate the effect size, for example, the difference in means, odds ratio, or relative risk ratio. No. 22(c). For full credit, authors must state precision as a confidence interval (CI). Only presenting *P*-values will score a “partial.”

No. 23. If additional analyses are prespecified in the research design, the results must be presented, *and* it must be clearly stated whether or not these were exploratory (e.g., post hoc) analyses.

No. 24. It cannot be scored as N/A. Authors need to state whether there were or were not adverse events or side effects for each group. If severe AEs occurred, estimates of the frequency should be reported.

No. 25(a). The relevance of the findings to the hypotheses or objectives must be discussed. No. 25(b). The strengths and weakness of the trial must be discussed, particularly in the context of potential for bias and imprecision of study findings. No. 25(c). This is usually scored as N/A unless numerous analyses both prespecified and exploratory were performed.

No. 26. “Generalizability” pertains to the external validity or general applicability of the results. For example, to what extent do the results of the study, with the population limited by the eligibility criteria, pertain to the greater population with this condition? Topics to consider are eligibility criteria, setting and location of the study, the intervention protocol, the period of recruitment and followup, and so forth.

No. 27. “General interpretation” pertains to how these results relate to previous research. For full credit, authors must (i) cite and (ii) discuss the previous research.

### B. Reference List of 333 RCTs Included in OCSI (Alphabetical)

1. Akimoto T, Nakahori C, Aizawa K, Kimura F, Fukubayashi T, Kono I. Acupuncture and Responses of Immunologic and Endocrine Markers during Competition. *Med Sci Sports Exerc* 2003; 35:1296–1302.
2. Alecrim-Andrade J, iel-Junior J, Cladellas X, Correa-Filho H, Machado H. Acupuncture in migraine prophylaxis: a randomized sham-controlled trial. *Cephalalgia* 2006; 26:520-529.
3. Alexander DN, Cen S, Sullivan KJ, Bhavnani G, Ma X, Azen SP. Effects of acupuncture treatment on poststroke motor recovery and physical function: a pilot study. *Neurorehabil Neural Repair* 2004; 18:259–267.
4. Alimi D, Rubino C, Pichard-Leandri E. Analgesic Effect of Auricular Acupuncture for Cancer Pain: A Randomized, Blinded, Controlled Trial. *J Clin Oncol* 2003; 21:4120–4126.
5. Allais G, De Lorenzo C, Quirico PE, Airola G, Tolardo G, Mana O et al. Acupuncture in the prophylactic treatment of migraine without aura: a comparison with flunarizine. *Headache* 2002; 42:855–861.
6. Allais G, De Lorenzo C, Quirico PE, Lupi G, Airola G, Mana O et al. Non-pharmacological approaches to chronic headaches: transcutaneous electrical nerve stimulation, lasertherapy and acupuncture in transformed migraine treatment. *Neurol Sci* 2003; 24:S138–S142.
7. Allen J, Schnyer R, Hitt S. The efficacy of acupuncture in the treatment of major depression in women. *Psych Science* 1998; 9:397–401.
8. Allen JJ, Schnyer RN, Chambers AS, Hitt SK, Moreno FA, Manber R. Acupuncture for depression: a randomized controlled trial. *J Clin Psychiatry* 2006; 67:1665–1673.
9. Alraek T, Soedal LI, Fagerheim SU, Digranes A, Baerheim A. Acupuncture treatment in the prevention of uncomplicated recurrent lower urinary tract infections in adult women. *Am J Public Health* 2002; 92:1609–1611.
10. Alraek T, Baerheim A. The effect of prophylactic acupuncture treatment in women with recurrent cystitis: kidney patients fare better. *J Altern Complement Med* 2003; 9:651–658.
11. Assefi NP, Sherman KJ, Jacobsen C, Goldberg J, Smith WR, Buchwald D. A randomized clinical trial of acupuncture compared with sham acupuncture in fibromyalgia. *Ann Intern Med* 2005; 143:10–19.
12. Aune A, Alraek T, LiHua H, Baerheim A. Acupuncture in the prophylaxis of recurrent lower urinary tract infection in adult women. *Scand J Prim Health Care* 1998; 16:37–39.
13. Avants SK, Margolin A, Holford TR, Kosten TR. A randomized controlled trial of auricular acupuncture for cocaine dependence. *Arch Intern Med* 2000; 160:2305–2312.
14. Bao YH, Feng W, zhu G, Zou C, Gong Y, Ji C et al. A Randomized and Comparative Study on Vascular Dementia Treated by Needling Remaining at Head Points. *EastWest Integrat Med* 2006; 4:12–17.
15. Bausell RB, Lao L, Bergman S, Lee WL, Berman BM. Is acupuncture analgesia an expectancy effect? Preliminary evidence based on participants' perceived assignments in two placebo-controlled trials. *Eval Health Prof* 2005; 28:9–26.
16. Berman AH, Lundberg U, Krook AL, Gyllenhammar C. Treating drug using prison inmates with auricular acupuncture; A randomized controlled trial. *J Subst Abuse Treat* 2004; 26:95–102.
17. Berman BM, Singh BB, Lao L, Langenberg P, Li H, Hadhazy V et al. A randomized trial of acupuncture as an adjunctive therapy in osteoarthritis of the knee. *Rheumatology (Oxford)* 1999; 38:346–354.
18. Berman BM, Lao L, Langenberg P, Lee WL, Gilpin AM, Hochberg MC. Effectiveness of acupuncture as adjunctive therapy in osteoarthritis of the knee: A randomized, controlled trial. *Ann Intern Med* 2004; 141:901–910.
19. Bier ID, Wilson J, Studt P, Shakleton M. Auricular acupuncture, education, and smoking cessation: a randomized, sham-controlled trial. *Am J Public Health* 2002; 92:1642–1647.
20. Biernacki W, Peake MD. Acupuncture in treatment of stable asthma. *Respir Med* 1998; 92:1143–1145.
21. Birch S, Jamison RN. Controlled trial of Japanese acupuncture for chronic myofascial neck pain: assessment of specific and nonspecific effects of treatment. *Clin J Pain* 1998; 14:248–255.
22. Bo Z, Wang G, Zhu W, Xiang Y, Yuan S, Niu Q. A clinical study on the therapeutic effect of abdominal acupuncture in treating radicular cervical spondylosis. *East West Integrat Med* 2007.
23. Brinkhaus B, Hummelsberger J, Kohnen R, Seufert J, Hempen CH, Leonhardy H et al. Acupuncture and Chinese herbal medicine in the treatment of patients with seasonal allergic rhinitis: a randomized-controlled clinical trial. *Allergy* 2004; 59:953–960.
24. Brinkhaus B, Witt CM, Jena S, Linde K, Streng A, Wagenpfeil S et al. Acupuncture in patients with chronic low back pain: a randomized controlled trial. *Arch Intern Med* 2006; 166:450–457.
25. Bu Y. Acupuncture Combined with Massage for Treatment of Cervical Spondylosis of Vertebral Artery Type. *East West Integrat Med* 2006; 4:44–46.
26. Bullock ML, Kiresuk TJ, Pheley AM, Culliton PD, Lenz SK. Auricular acupuncture in the treatment of cocaine abuse. A study of efficacy and dosing. *J Subst Abuse Treat* 1999; 16:31–38.
27. Bullock ML, Kiresuk TJ, Sherman RE, Lenz SK, Culliton PD, Boucher TA et al. A large randomized placebo controlled study of auricular acupuncture for alcohol dependence. *J Subst Abuse Treat* 2002; 22:71–77.
28. Cabrini L, Gioia L, Gemma M, Melloni G, Carretta A, Ciriaco P et al. Acupuncture for diagnostic fiberoptic bronchoscopy: a prospective, randomized, placebo-controlled study. *Am J Chin Med* 2006; 34:409–415.
29. Cao J, Qu F, Zhou J. Improvement of Gastic Movementon Patients with Functionsal Dyspepsia Treated with Acupuncture. *Intl J Clin Acu* 2007; 16:23–26.
30. Carlsson CP, Axemo P, Bodin A et al. Manual acupuncture reduces hyperemesis gravidarum: A placebo-controlled, randomized, single-blind, cross-over study. *J Pain Symptom Manage* 2000; 20:273–279.
31. Carlsson CP, Sjolund BH. Acupuncture for chronic low back pain: a randomized placebo-controlled study with long-term follow-up. *Clin J Pain* 2001; 17:296–305.
32. Chen CH, Chen TW, Weng MC, Wang WT, Wang Yl, Huang MH. The effect of electroacupuncture on shoulder subluxation for stroke patients. *Kaohsiung J Med Sci* 2000; 16:525–532.
33. Cheng PT, Wong MK, Chang PL. A therapeutic trial of acupuncture in neurogenic bladder of spinal cord injured patients–a preliminary report. *Spinal Cord* 1998; 36:476–480.
34. Cherkin DC, Eisenberg D, Sherman KJ, Barlow W, Kaptchuk TJ, Street J et al. Randomized trial comparing traditional Chinese medical acupuncture, therapeutic massage, and self-care education for chronic low back pain. *Arch Intern Med* 2001; 161:1081–1088.
35. Chu KA, Wu YC, Ting YM, Wang HC, Lu JY. Acupuncture therapy results in immediate bronchodilating effect in asthma patients. *J Chin Med Assoc* 2007; 70:265–268.
36. Cittadini M, Marmori F, Diacinti D, Walker JI. Randomized Trial of Acupuncture Compared with Prokinetic Drugs and Sham Acupuncture for Chronic Idiopathic Dyspepsia. *Med Acupunct* 2003; 14:17-18.
37. Coe T. The Effect of Acupuncture on Pain and Swelling after Day Case Molar Teeth extraction Under General Anaesthesia. *Ambulatory Surg* 1999; 7:45–49.
38. Coeytaux RR, Kaufman JS, Kaptchuk TJ, Chen W, Miller WC, Callahan LF et al. A randomized, controlled trial of acupuncture for chronic daily headache. *Headache* 2005; 45:1113–1123.
39. Cohen SM, Rousseau ME, Carey BL. Can acupuncture ease the symptoms of menopause? *Holist Nurs Pract* 2003; 17:295–299.
40. Cristian A, Katz M, Cutrone E, Walker RH. Evaluation of acupuncture in the treatment of Parkinson's disease: a double-blind pilot study. *Mov Disord* 2005; 20:1185–1188.
41. Cui R, Zhou D. Treatment of phlegm- and heat-induced insomnia by acupuncture in 120 cases. *J Tradit Chin Med* 2003; 23:57-58.
42. Dang W, Yang J. Clinical study on acupuncture treatment of stomach carcinoma pain. *J Tradit Chin Med* 1998; 18:31–38.
43. David J, Modi S, Aluko AAea. Chronic neck pain: A comparison of acupuncture treatment and physiotherapy. *Brit J Rheumatol* 1998; 37:1118–1112.
44. David J, Townsend S, Sathanathan R, Kriss S, Dore CJ. The effect of acupuncture on patients with rheumatoid arthritis: a randomized, placebo-controlled cross-over study. *Rheumatology (Oxford)* 1999; 38:864–869.
45. Deng WYZ. Clinical Observation of Treating Migraine with Deep Needling at Point Fengchi. *Intl J Clin Acu* 2006; 15:95–98.
46. Dickman R, Schiff E, Holland A, Wright C, Sarela SR, Han B et al. Acupuncture vs. doubling the PPI dose in refractory heartburn. *Aliment Pharmacol Ther* 2007.
47. Diener HC, Kronfeld K, Boewing G, Lungenhausen M, Maier C, Molsberger A et al. Efficacy of acupuncture for the prophylaxis of migraine: a multicentre randomised controlled clinical trial. *Lancet Neurol* 2006; 5:310–316.
48. Dieterle S, Ying G, Hatzmann W, Neuer A. Effect of acupuncture on the outcome of in vitro fertilization and intracytoplasmic sperm injection: a randomized, prospective, controlled clinical study. *Fertil Steril* 2006.
49. Ding X, Gao Q. Acupuncture treatment for multiple aortitis–a clinical report of 40 cases. *J Tradit Chin Med* 2005; 25:260–263.
50. Du G. Clinical research on treatment of premonitory apoplexy with electro-acupuncture of head points. *Int J Clin Acupunct* 2001; 12:15–20.
51. Duncan B, Barton L, Edmonds D, Blashill BM. Parental perceptions of the therapeutic effect from osteopathic manipulation or acupuncture in children with spastic cerebral palsy. *Clin Pediatr (Phila)* 2004; 43:349–353.
52. Dyrehag LE, Widerstrom-Noga EG, Carlsson SG, Andersson SA. Effects of repeated sensory stimulation sessions (electro-acupuncture) on skin temperature in chronic pain patients. *Scand J Rehabil Med* 1997; 29:243–250.
53. Dyson-Hudson TA, Shiflett SC, Kirshblum SC, Bowen JE, Druin EL. Acupuncture and Trager psychophysical integration in the treatment of wheelchair user's shoulder pain in individuals with spinal cord injury. *Arch Phys Med Rehabil* 2001; 82:1038–1046.
54. Elden H, Ladfors L, Olsen MF, Ostgaard HC, Hagberg H. Effects of acupuncture and stabilising exercises as adjunct to standard treatment in pregnant women with pelvic girdle pain: randomised single blind controlled trial. *BMJ* 2005; 330:761.
55. Endres HG, Bowing G, Diener HC, Lange S, Maier C, Molsberger A et al. Acupuncture for tension-type headache: a multicentre, sham-controlled, patient-and observer-blinded, randomised trial. *J Headache Pain* 2007; 8:306–314.
56. Engelhardt PF, Daha LK, Zils T, Simak R, Konig K, Pfluger H. Acupuncture in the treatment of psychogenic erectile dysfunction: first results of a prospective randomized placebo-controlled study. *Int J Impot Res* 2003; 15:343–346.
57. Fanti L, Gemma M, Passaretti S, Guslandi M, Testoni PA, Casati A et al. Electroacupuncture analgesia for colonoscopy. a prospective, randomized, placebo-controlled study. *Am J Gastroenterol* 2003; 98:312–316.
58. Fink M, Wolkenstein E, Karst M, Gehrke A. Acupuncture in chronic epicondylitis: a randomized controlled trial. *Rheumatology (Oxford)* 2002; 41:205–209.
59. Fink M, Rollnik JD, Bijak M, Borstadt C, Dauper J, Guergueltcheva V et al. Needle acupuncture in chronic poststroke leg spasticity. *Arch Phys Med Rehabil* 2004; 85:667–672.
60. Fink MG, Wipperman B, Gehrke A. Non-specific effects of traditional Chinese acupuncture in osteoarthritis of the hip. *Complement Ther Med* 2001; 9:82–89.
61. Fireman Z, Segal A, Kopelman Y, Sternberg A, Carasso R. Acupuncture treatment for irritable bowel syndrome. A double-blind controlled study. *Digestion* 2001; 64:100–103.
62. Flachskampf FA, Gallasch J, Gefeller O, Gan J, Mao J, Pfahlberg AB et al. Randomized trial of acupuncture to lower blood pressure. *Circulation* 2007; 115:3121–3129.
63. Forbes A, Jackson S, Walter C, Quraishi S, Jacyna M, Pitcher M. Acupuncture for irritable bowel syndrome: A blinded placebo-controlled trial. *World J Gastroenterol* 2005; 11:4040–4044.
64. Foster NE, Thomas E, Barlas P, Hill JC, Young J, Mason E et al. Acupuncture as an adjunct to exercise based physiotherapy for osteoarthritis of the knee: randomised controlled trial. *BMJ* 2007; 335:436.
65. Freire AO, Sugai GC, Chrispin FS, Togeiro SM, Yamamura Y, Mello LE et al. Treatment of moderate obstructive sleep apnea syndrome with acupuncture: a randomised, placebo-controlled pilot trial. *Sleep Med* 2007; 8:43–50.
66. Fu B, Lun X, Gong Y. Effects of the combined therapy of acupuncture with herbal drugs on male immune infertility–a clinical report of 50 cases. *J Tradit Chin Med* 2005; 25:186–189.
67. Gallagher SM, Allen JJ, Hitt SK, Schnyer RN, Manber R. Six-month depression relapse rates among women treated with acupuncture. *Complement Ther Med* 2001; 9:216–218.
68. Gao H, Yan L, Liu B, Wang Y, Wei X, Sun L et al. Clinical study on treatment of senile vascular dementia by acupuncture. *J Tradit Chin Med* 2001; 21:103–109.
69. Gao H, Zhang W, Wang Y. Acupuncture treatment for 34 cases of uremic cutaneous pruritus. *J Tradit Chin Med* 2002; 22:29–30.
70. Gao S, Zhao D, Xie Y. A comparative study on the treatment of migraine headache with combined distant and local acupuncture points versus conventional drug therapy. *Am J Acupunct* 1999; 27:27–30.
71. Gaudernack LC, Forbord S, Hole E. Acupuncture administered after spontaneous rupture of membranes at term significantly reduces the length of birth and use of oxytocin. A randomized controlled trial. *Acta Obstet Gynecol Scand* 2006; 85:1348–1353.
72. Gibson D, Bruton A, Lewith GT, Mullee M. Effects of acupuncture as a treatment for hyperventilation syndrome: a pilot, randomized crossover trial. *J Altern Complement Med* 2007; 13:39–46.
73. Gilbertson B, Wenner K, Russell LC. Acupuncture and arthroscopic acromioplasty. *J Orthop Res* 2003; 21:752–758.
74. Giles LG, Muller R. Chronic spinal pain syndromes: a clinical pilot trial comparing acupuncture, a nonsteroidal anti-inflammatory drug, and spinal manipulation. *J Manipulative Physiol Ther* 1999; 22:376–381.
75. Giles LG, Muller R. Chronic Spinal Pain: A Randomized Clinical Trial Comparing Medication, Acupuncture, and Spinal Manipulation. *Spine* 2003; 28:1490–1502.
76. Gioia L, Cabrini L, Gemma M, Fiori R, Fasce F, Bolognesi G et al. Sedative effect of acupuncture during cataract surgery: prospective randomized double-blind study. *J Cataract Refract Surg* 2006; 32:1951–1954.
77. Goddard G, Karibe H, McNeill C, Villafuerte E. Acupuncture and sham acupuncture reduce muscle pain in myofascial pain patients. *J Orofac Pain* 2002; 16:71–76.
78. Goertz CM, Niemtzow R, Burns SM, Fritts MJ, Crawford CC, Jonas WB. Auricular acupuncture in the treatment of acute pain syndromes: A pilot study. *Mil Med* 2006; 171:1010–1014.
79. Gosman-Hedstrom G. Effects of acupuncture treatment on daily life activities and quality of life: A controlled, prospective, and randomized study of acute stroke patients. *Stroke* 1998; 29:2100–2108.
80. Grant DJ, Bishop-Miller J, Winchester DM, Anderson M, Faulkner S. A randomized comparative trial of acupuncture versus transcutaneous electrical nerve stimulation for chronic back pain in the elderly. *Pain* 1999; 82:9–13.
81. Gronlund MA, Stenevi U, Lundeberg T. Acupuncture treatment in patients with keratoconjunctivitis sicca: a pilot study. *Acta Ophthalmol Scand* 2004; 82:283–290.
82. Guerra de Hoyos JA, Martin MC, Leon EB, Lopez MV, Lopez TM, Morilla FA et al. Randomised trial of long term effect of acupuncture for shoulder pain. *Pain* 2004; 112:289–298.
83. Guerreiro da Silva JB, Nakamura MU, Cordeiro JA, Kulay L, Jr. Acupuncture for low back pain in pregnancy–a prospective, quasi-randomised, controlled study. *Acupunct Med* 2004; 22:60–67.
84. Guo F. Observation on Treatment of Dizziness Mainly by Acupuncture. *J Trad Chin Med* 2007; 27:16–18.
85. Guo Z. Forty-six cases of Acute Cerebral Infartion Treated with the Combined use of Acupuncture and Drugs. *J Trad Chin Med* 2007; 27:243–247.
86. Gupta S, Francis JD, Tillu AB, Sattirajah AI, Sizer J. The effect of pre-emptive acupuncture treatment on analgesic requirements after day-case knee arthroscopy. *Anaesthesia* 1999; 54:1204–1207.
87. Gurfinkel E, Cedenho AP, Yamamura Y, Srougi M. Effects of acupuncture and moxa treatment in patients with semen abnormalities. *Asian J Androl* 2003; 5:345–348.
88. Habek D, Habek JC, Barbir A. Using acupuncture to treat premenstrual syndrome. *Arch Gynecol Obstet* 2002; 267:23–26.
89. Habek D, Cerkez HJ, Jagust M. Acupuncture conversion of fetal breech presentation. *Fetal Diagn Ther* 2003; 18:418–421.
90. Habek D, Barbir A, Habek JC, Janculiak D, Bobic-Vukovic M. Success of acupuncture and acupressure of the Pc 6 acupoint in the treatment of hyperemesis gravidarum. *Forsch Komplementarmed Klass Naturheilkd* 2004; 11:20–23.
91. Han S, Li C. Acupuncture Treatment for 68 Cases of Functional Impairment Induced by Cerebral Hemmorage at the Convalescence Stage. *J TCM* 2006; 26:172–174.
92. Hantoushzadeh S, Alhusseini N, Lebaschi AH. The effects of acupuncture during labour on nulliparous women: A randomised controlled trial. *Aust N Z J Obstet Gynaecol* 2007; 47:26–30.
93. Harper TC, Coeytaux RR, Chen W, Campbell K, Kaufman JS, Moise KJ et al. A randomized controlled trial of acupuncture for initiation of labor in nulliparous women. *J Matern Fetal Neonatal Med* 2006; 19:465–470.
94. Harris RE, Tian X, Williams DA, Tian TX, Cupps TR, Petzke F et al. Treatment of fibromyalgia with formula acupuncture: investigation of needle placement, needle stimulation, and treatment frequency. *J Altern Complement Med* 2005; 11:663–671.
95. Haslam R. A comparison of acupuncture with advice and exercises on the symptomatic treatment of osteoarthritis of the hip–a randomised controlled trial. *Acupunct Med* 2001; 19:19–26.
96. He D, Medbo JI, Hostmark AT. Effect of acupuncture on smoking cessation or reduction: an 8-month and 5-year follow-up study. *Prev Med* 2001; 33:364–372.
97. He D, Bo VK, Hostmark AT, Ingulf MJ. Effect of acupuncture treatment on chronic neck and shoulder pain in sedentary female workers: a 6-month and 3-year follow-up study. *Pain* 2004; 109:299–307.
98. He D, Hostmark AT, Veiersted KB, Medbo JI. Effect of intensive acupuncture on pain-related social and psychological variables for women with chronic neck and shoulder pain–an RCT with six month and three year follow up. *Acupunct Med* 2005; 23:52–61.
99. He J, Wu B, Zhang Y. Acupuncture treatment for 15 cases of post-traumatic coma. *J Tradit Chin Med* 2005; 25:171–173.
100. Hollifield M, Sinclair-Lian N, Warner TD, Hammerschlag R. Acupuncture for posttraumatic stress disorder: a randomized controlled pilot trial. *J Nerv Ment Dis* 2007; 195:504–513.
101. Hsieh RL, Wang LY, Lee WC. Additional therapeutic effects of electroacupuncture in conjunction with conventional rehabilitation for patients with first-ever ischaemic stroke. *J Rehabil Med* 2007; 39:205–211.
102. Hsu Ch, Hwang KC, Chao CL, Lin JG, Kao ST, Chou P. Effects of electroacupuncture in reducing weight and waist circumference in obese women: a randomized crossover trial. *Int J Obes (Lond)* 2005; 29:1379–1384.
103. Huang MI, Nir Y, Chen B, Schnyer R, Manber R. A randomized controlled pilot study of acupuncture for postmenopausal hot flashes: effect on nocturnal hot flashes and sleep quality. *Fertil Steril* 2006; 86:700–710.
104. Huguenin L, Brukner PD, McCrory P, Smith P, Wajswelner H, Bennell K. Effect of dry needling of gluteal muscles on straight leg raise: a randomised, placebo controlled, double blind trial. *Br J Sports Med* 2005; 39:84–90.
105. Humaidan P, Stener-Victorin E. Pain relief during oocyte retrieval with a new short duration electro-acupuncture technique–an alternative to conventional analgesic methods. *Hum Reprod* 2004.
106. Inoue M, Kitakoji H, Ishizaki N, Tawa M, Yano T, Katsumi Y et al. Relief of low back pain immediately after acupuncture treatment - a randomised, placebo controlled trial. *Acupunct Med* 2006; 24:103–108.
107. Irnich D, Behrens N, Molzen H, Konig A, Gleditsch J, Krauss M et al. Randomised trial of acupuncture compared with conventional massage and "sham" laser acupuncture for treatment of chronic neck pain. * BMJ* 2001; 322:1574–1578.
108. Irnich D, Behrens N, Gleditsch J, Stor W, Schreiber M, Schops P et al. Immediate effects of dry needling and acupuncture at distant points in chronic neck pain: results of a randomized, double-blind, sham-controlled crossover trial. *Pain* 2002; 99:83.
109. Itoh K, Katsumi Y, Hirota S, Kitakoji H. Effects of trigger point acupuncture on chronic low back pain in elderly patients–a sham-controlled randomised trial. *Acupunct Med* 2006; 24:5–12.
110. Jensen R, Gothesen O, Liseth K, Baerheim A. Acupuncture treatment of patellofemoral pain syndrome. *J Altern Complement Med* 1999; 5:521–527.
111. Jiang H, Shi K, Li X, Zhou W, Cao Y. Clinical study on the wrist-ankle acupuncture treatment for 30 cases of diabetic peripheral neuritis. *J Tradit Chin Med* 2006; 26:8–12.
112. Jiang Z, Li C, Li Y. Treatment of postapoplectic thalamic spontaneous pain by electroacupuncture at huatuojiaji points. *J Tradit Chin Med* 1999; 19:195–199.
113. Jiang Zy, Li Cd, Li Y. Migraine treated by acupuncture. *Int J Clin Acupunct* 2000; 11:265–268.
114. Jiang Zy, Li Cd. Needling paravertebral points in treatment of post-stroke thalamic pain. *Int J Clin Acupunct* 2000; 11:7–10.
115. Johansson BB, Haker E, von Arbin M, Britton M, Langstrom G, Terent A et al. Acupuncture and transcutaneous nerve stimulation in stroke rehabilitation: a randomized, controlled trial. *Stroke* 2001; 32:707–713.
116. Johansson KM, Adolfsson LE, Foldevi MO. Effects of acupuncture versus ultrasound in patients with impingement syndrome: randomized clinical trial. *Phys Ther* 2005; 85:490–501.
117. Johnstone PA, Bloom TL, Niemtzow RC, Crain D, Riffenburgh RH, Amling CL. A Prospective, Randomized Pilot Trial of Acupuncture of the Kidney-Bladder Distinct Meridian for Lower Urinary Tract Symptoms. *J Urol* 2003; 169:1037–1039.
118. Joos S, Schott C, Zou H, Daniel V, Martin E. Immunomodulatory effects of acupuncture in the treatment of allergic asthma: a randomized controlled study. *J Altern Complement Med* 2000; 6:519–525.
119. Joos S, Brinkhaus B, Maluche C, Maupai N, Kohnen R, Kraehmer N et al. Acupuncture and Moxibustion in the Treatment of Active Crohn's Disease: A Randomized Controlled Study. *Digestion* 2004; 69:131–139.
120. Joos S, Wildau N, Kohnen R, Szecsenyi J, Schuppan D, Willich SN et al. Acupuncture and moxibustion in the treatment of ulcerative colitis: A randomized controlled study. *Scand J Gastroenterol* 2006; 41:1056–1063.
121. Karst M, Rollnik JD, Fink M, Reinhard M, Piepenbrock S. Pressure pain threshold and needle acupuncture in chronic tension-type headache–a double-blind placebo-controlled study. *Pain* 2000; 88:199–203.
122. Karst M, Reinhard M, Thum P, Wiese B, Rollnik J, Fink M. Needle acupuncture in tension-type headache: a randomized, placebo-controlled study. *Cephalalgia* 2001; 21:637–642.
123. Karst M, Passie T, Friedrich S, Wiese B, Schneider U. Acupuncture in the treatment of alcohol withdrawal symptoms: a randomized, placebo-controlled inpatient study. *Addict Biol* 2002; 7:415–419.
124. Karst M, Winterhalter M, Munte S, Francki B, Hondronikos A, Eckardt A et al. Auricular acupuncture for dental anxiety: a randomized controlled trial. *Anesth Analg* 2007; 104:295–300.
125. Kawakita K, Shichidou T, Inoue E, Nabeta T, Kitakouji H, Aizawa S et al. Preventive and curative effects of acupuncture on the common cold: a multicentre randomized controlled trial in Japan. *Complement Ther Med* 2004; 12:181–188.
126. Kerr DP, Walsh DM, Baxter D. Acupuncture in the management of chronic low back pain: a blinded randomized controlled trial. *Clin J Pain* 2003; 19:364–370.
127. Khoo KK. Acupuncture treatment for obesity: a randomized controlled trial. *Med Acupunct* 2006; 17:33–35.
128. Killeen TK, Haight B, Brady K, Herman J, Michel Y, Stuart G et al. The effect of auricular acupuncture on psychophysiological measures of cocaine craving. *Issues Ment Health Nurs* 2002; 23:445–459.
129. Kim Y, Kim CW, Kim KS. Clinical observations on postoperative vomiting treated by auricular acupuncture. *Am J Chin Med* 2003; 31:475–480.
130. Kim YS, Lee SH, Jung WS, Park SU, Moon SK, Ko CN et al. Intradermal acupuncture on shen-men and nei-kuan acupoints in patients with insomnia after stroke. *Am J Chin Med* 2004; 32:771–778.
131. Kitade T, Ohyabu H. Analgesic effects of acupuncture on pain after mandibular wisdom tooth extraction. *Acupunct Electrother Res* 2000; 25:109–115.
132. Kleinhenz J, Streitberger K, Windeler J, Gussbacher A, Mavridis G, Martin E. Randomised clinical trial comparing the effects of acupuncture and a newly designed placebo needle in rotator cuff tendinitis. *Pain* 1999; 83:235–241.
133. Kloster R, Larsson PG, Lossius R, Nakken KO, Dahl R, Xiu-Ling X et al. The effect of acupuncture in chronic intractable epilepsy. *Seizure* 1999; 8:170–174.
134. Knight B, Mudge C, Openshaw S, White A, Hart A. Effect of acupuncture on nausea of pregnancy: a randomized, controlled trial. *Obstet Gynecol* 2001; 97:184–188.
135. Korpan MI, Dezu Y, Schneider B, Leitha T, Fialka-Moser V. Acupuncture in the treatment of posttraumatic pain syndrome. *Acta Orthop Belg* 1999; 65:197–201.
136. Kvist LJ, Wilde LB, Hall-Lord ML, Rydhstroem H. Effects of acupuncture and care interventions on the outcome of inflammatory symptoms of the breast in lactating women. *Int Nurs Rev* 2004; 51:56–64.
137. Kvorning N, Christiansson C, Beskow A, Bratt O, Akeson J. Acupuncture fails to reduce but increases anaesthetic gas required to prevent movement in response to surgical incision. *Acta Anaesthesiol Scand* 2003; 47:818–822.
138. Kvorning N, Christiansson C, Akeson J. Acupuncture facilitates neuromuscular and oculomotor responses to skin incision with no influence on auditory evoked potentials under sevoflurane anaesthesia. *Acta Anaesthesiol Scand* 2003; 47:1073–1078.
139. Kvorning N, Holmberg C, Grennert L, Aberg A, Akeson J. Acupuncture relieves pelvic and low-back pain in late pregnancy. *Acta Obstet Gynecol Scand* 2004; 83:246–250.
140. Lao L, Bergman S, Hamilton GR, Langenberg P, Berman B. Evaluation of acupuncture for pain control after oral surgery: a placebo-controlled trial. *Arch Otolaryngol Head Neck Surg* 1999; 125:567–572.
141. Leibing E, Leonhardt U, Koster G, Goerlitz A, Rosenfeldt JA, Hilgers R et al. Acupuncture treatment of chronic low-back pain – a randomized, blinded, placebo-controlled trial with 9-month follow-up. *Pain* 2002; 96:189–196.
142. Lewith GT, Prescott P, Davis CL. Can a standardized acupuncture technique palliate disabling breathlessness: a single-blind, placebo-controlled crossover study. *Chest* 2004; 125:1783–1790.
143. Li H, Wang J, Lu G, Zhang Q. Effect of an Herbal Compound Combined with Acupuncture and Qigong on Angina Pectoris. *Am J Tradit Chin Med* 2006; 7:8–13.
144. Li J. Clinical study on effect of scalp-acupuncture in treating acute cerebral hemorrhage. *Chin J Integrat Trad West Med* 1999; 5:265–268.
145. Li Y, Liang FR, Yu SG, Li Cd, Hu LX, Zhou D et al. Efficacy of acupuncture and moxibustion in treating Bell's palsy: a multicenter randomized controlled trial in China. *Chin Med J (Engl )* 2004; 117:1502–1506.
146. Liang F, Li Y, Yu S, Li C, Hu L, Zhou D et al. A multicentral randomized control study on clinical acupuncture treatment of Bell's palsy. *J Tradit Chin Med* 2006; 26:3–7.
147. Liguori A, Petti F, Bangrazi A, Camaioni D, Guccione G, Pitari GM et al. Comparison of pharmacological treatment versus acupuncture treatment for migraine without aura–analysis of socio-medical parameters. *J Tradit Chin Med* 2000; 20:231–240.
148. Lin H, Li C. Clinical observation on treatment of auditory hallucinosis by electroacupuncture: a report of 30 cases. *J Tradit Chin Med* 2005; 25:102–103.
149. Lin JG, Lo MW, Wen YR, Hsieh CL, Tsai SK, Sun WZ. The effect of high and low frequency electroacupuncture in pain after lower abdominal surgery. *Pain* 2002; 99:509–514.
150. Lin Q, Li X, Han J, Leng J. Electro-acupuncture treatment for the upper segment ureterolithiasis under B-ultrasonography. *J Tradit Chin Med* 2005; 25:13–15.
151. Linde K, Streng A, Jurgens S, Hoppe A, Brinkhaus B, Witt C et al. Acupuncture for patients with migraine: a randomized controlled trial. *JAMA* 2005; 293:2118–2125.
152. Linde M, Fjell A, Carlsson J, Dahlof C. Role of the needling per se in acupuncture as prophylaxis for menstrually related migraine: a randomized placebo-controlled study. *Cephalalgia* 2005; 25:41–47.
153. Linde MA, Carlsson J, Dahlof C. Impact of acupuncture as add-on therapy to pharmacological treatment of migraine: A pilot study. *Pain Clinic* 2000; 12:247–252.
154. List T, Lundeberg T, Lundstrom I, Lindstrom F, Ravald N. The effect of acupuncture in the treatment of patients with primary Sjogren's syndrome. A controlled study. *Acta Odontol Scand* 1998; 56:95–99.
155. Liu G, Zang Y, Guo L. Comparative study on Acupuncture Combined with Behavioral Desensitization for Treatment of Anxiety Neuroses. *Am J Acupunct* 1998; 26:117–120.
156. Liu Y, Zhang L. The TCM-combined treatment for aphasia due to cerebrovascular disorders. *EastWest Integrat Med* 2006; 22–24.
157. Liu Z, Liu B, Yang T, Ye Y, Zhao H, Zhang W et al. Clinical study of Electroacupuncture Treatment of Senile Urge Urinary Incontinence. *Int J Clin Acupunct* 2002; 13:255–262.
158. Lu DP, Lu GP, Reed JF, III. Acupuncture/acupressure to treat gagging dental patients: a clinical study of anti-gagging effects. *Gen Dent* 2000; 48:446–452.
159. Lun X, Rong L. Twenty-five cases of intractable cutaneous pruritus treated by auricular acupuncture. *J Tradit Chin Med* 2000; 20:287–288.
160. Lun X, Yang W, Fu B. Effects of CT-Localized Scalp Round-Needling on the Blood Rheology, NO and NOS of Patients with Mukltiple Infarctional Dementia. *J Trad Chin Med* 2006; 26:92–96.
161. Luo H, Meng F, Jia Y, Zhao X. Clinical research on the therapeutic effect of the electro-acupuncture treatment in patients with depression. *Psychiatry Clin Neurosci* 1998; 52 Suppl: S338–S340.
162. Ma Rh, Sha Ge, Liu Xm. Acupuncture Treatment of Amenorrhea due to Medication: Clinical Observation of 117 Cases. *Int J Clin Acupunct* 1999; 10:105–109.
163. Ma T, Kao MJ, Lin IH, Chiu YL, Chien C, Ho TJ et al. A study on the clinical effects of physical therapy and acupuncture to treat spontaneous frozen shoulder. *Am J Chin Med* 2006; 34:759–775.
164. Ma X. Clinical analysis for the acupuncture treatment in 42 cases of gouty renal damage. *J Tradit Chin Med* 2004; 24:185–187.
165. Maa SH, Sun MF, Hsu KH, Hung TJ, Chen HC, Yu CT et al. Effect of acupuncture or acupressure on quality of life of patients with chronic obstructive asthma: a pilot study. *J Altern Complement Med* 2003; 9:659–670.
166. Macklin EA, Wayne PM, Kalish LA, Valaskatgis P, Thompson J, Pian-Smith MC et al. Stop Hypertension with the Acupuncture Research Program (SHARP): results of a randomized, controlled clinical trial. *Hypertension* 2006; 48:838–845.
167. Magnusson AL, Svensson RE, Leirvik C, Gunnarsson RK. The effect of acupuncture on allergic rhinitis: a randomized controlled clinical trial. *Am J Chin Med* 2004; 32:105–115.
168. Malmstrom M, Ahlner J, Carlsson C, Schmekel B. No effect of chinese acupuncture on isocapnic hyperventilation with cold air in asthmatics, measured with impulse oscillometry. *Acupunct Med* 2002; 20:66–73.
169. Manber R, Schnyer RN, Allen JJ, Rush AJ, Blasey CM. Acupuncture: a promising treatment for depression during pregnancy. *J Affect Disord* 2004; 83:89–95.
170. Margolin A, Avants SK, Chang PL. Acupuncture for the Treatment of Cocaine Dependence in Methadone-Maintained Patients. *Am J Addic* 1993; 2:194–201.
171. Martin DP, Sletten CD, Williams BA, Berger IH. Improvement in fibromyalgia symptoms with acupuncture: results of a randomized controlled trial. *Mayo Clin Proc* 2006; 81:749–757.
172. Mazzoni R, Mannucci E, Rizzello SM, Ricca V, Rotella CM. Failure of acupuncture in the treatment of obesity: a pilot study. *Eat Weight Disord* 1999; 4:198–202.
173. Medici TC, Grebski E, Wu J, Hinz G, Wuthrich B. Acupuncture and bronchial asthma: a long-term randomized study of the effects of real versus sham acupuncture compared to controls in patients with bronchial asthma. *J Altern Complement Med* 2002; 8:737–750.
174. Mehl-Madrona L, Kligler B, Silverman S, Lynton H, Merrell W. The impact of acupuncture and craniosacral therapy interventions on clinical outcomes in adults with asthma. *Explore (NY)* 2007; 3:28–36.
175. Mehling WE, Jacobs B, Acree M, Wilson L, Bostrom A, West J et al. Symptom management with massage and acupuncture in postoperative cancer patients: a randomized controlled trial. *J Pain Symptom Manage* 2007; 33:258–266.
176. Melchart D, Thormaehlen J, Hager S, Liao J, Linde K, Weidenhammer W. Acupuncture versus placebo versus sumatriptan for early treatment of migraine attacks: a randomized controlled trial. *J Intern Med* 2003; 253:181–188.
177. Melchart D, Hager S, Hager U, Liao J, Weidenhammer W, Linde K. Treatment of patients with chronic headaches in a hospital for traditional Chinese medicine in Germany. A randomised, waiting list controlled trial. *Complement Ther Med* 2004; 12:71–78.
178. Melchart D, Streng A, Hoppe A, Brinkhaus B, Witt C, Wagenpfeil S et al. Acupuncture in patients with tension-type headache: randomised controlled trial. *BMJ* 2005; 331:376–382.
179. Melchart D, Ihbe-Heffinger A, Leps B, von SC, Linde K. Acupuncture and acupressure for the prevention of chemotherapy-induced nausea-a randomised cross-over pilot study. *Support Care Cancer* 2006; 14:878–882.
180. Meng CF, Wang D, Ngeow J, Lao L, Peterson M, Paget S. Acupuncture for chronic low back pain in older patients: a randomized, controlled trial. *Rheumatology (Oxford)* 2003; 42:1508–1517.
181. Meng J. The effects of acupuncture in treatment of coronary heart diseases. *J Tradit Chin Med* 2004; 24:16–19.
182. Michalek-Sauberer A, Heinzl H, Sator-Katzenschlager SM, Monov G, Knolle E, Kress HG. Perioperative auricular electroacupuncture has no effect on pain and analgesic consumption after third molar tooth extraction. *Anesth Analg* 2007; 104:542–547.
183. Middlekauff HR, Hui K, Yu JL, Hamilton MA, Fonarow GC, Moriguchi J et al. Acupuncture inhibits sympathetic activation during mental stress in advanced heart failure patients. *J Card Fail* 2002; 8:399–406.
184. Millea PJ, Reed B. Acupuncture in the treatment of alzheimer disease complicated by agitation: A randomized controlled pilot study. *Med Acupunct* 2004; 15:19–23.
185. Molsberger AF, Mau J, Pawelec DB, Winkler J. Does acupuncture improve the orthopedic management of chronic low back pain - a randomized, blinded, controlled trial with 3 months follow up. *Pain* 2002; 99:579–587.
186. Montazeri K, Farahnakian M, Saghaei M. The effect of acupuncture on the acute withdrawal symptoms from rapid opiate detoxification. *Acta Anaesthesiol Sin* 2002; 40:173–177.
187. Mukherjee M, McPeak LK, Redford JB, Sun C, Liu W. The effect of electro-acupuncture on spasticity of the wrist joint in chronic stroke survivors. *Arch Phys Med Rehabil* 2007; 88:159–166.
188. Nabeta T, Kawakita K. Relief of chronic neck and shoulder pain by manual acupuncture to tender points-a sham-controlled randomized trial. *Complement Ther Med* 2003; 10:217–222.
189. Naslund J, Naslund UB, Odenbring S, Lundeberg T. Sensory stimulation (acupuncture) for the treatment of idiopathic anterior knee pain. *J Rehabil Med* 2002; 34:231–238.
190. Nedstrand E, Wyon Y, Hammar M, Wijma K. Psychological well-being improves in women with breast cancer after treatment with applied relaxation or electro-acupuncture for vasomotor symptom. *J Psychosom Obstet Gynaecol* 2006; 27:193–199.
191. Neri I, Airola G, Contu G, Allais G, Facchinetti F, Benedetto C. Acupuncture plus moxibustion to resolve breech presentation: a randomized controlled study. *J Matern Fetal Neonatal Med* 2004; 15:247–252.
192. Neri I, Allais G, Schiapparelli P, Blasi I, Benedetto C, Facchinetti F. Acupuncture versus pharmacological approach to reduce Hyperemesis gravidarum discomfort. *Minerva Ginecol* 2005; 57:471–475.
193. Nesheim BI, Kinge R, Berg B, Alfredsson B, Allgot E, Hove G et al. Acupuncture during labor can reduce the use of meperidine: a controlled clinical study. *Clin J Pain* 2003; 19:187–191.
194. Ng DK, Chow PY, Ming SP, Hong SH, Lau S, Tse D et al. A double-blind, randomized, placebo-controlled trial of acupuncture for the treatment of childhood persistent allergic rhinitis. *Pediatrics* 2004; 114:1242–1247.
195. Ng MM, Leung MC, Poon DM. The effects of electro-acupuncture and transcutaneous electrical nerve stimulation on patients with painful osteoarthritic knees: a randomized controlled trial with follow-up evaluation. *J Altern Complement Med* 2003; 9:641–649.
196. Nir Y, Huang MI, Schnyer R, Chen B, Manber R. Acupuncture for postmenopausal hot flashes. *Maturitas* 2006.
197. Otto KC, Quinn C, Sung YF. Auricular acupuncture as an adjunctive treatment for cocaine addiction. A pilot study. *Am J Addict* 1998; 7:164–170.
198. Paraskeva A, Melemeni A, Petropoulos G, Siafaka I, Fassoulaki A. Needling of the extra 1 point decreases BIS values and preoperative anxiety. *Am J Chin Med* 2004; 32:789–794.
199. Park J, White AR, James MA, Hemsley AG, Johnson P, Chambers J et al. Acupuncture for subacute stroke rehabilitation: a Sham-controlled, subject- and assessor-blind, randomized trial. *Arch Intern Med* 2005; 165:2026–2031.
200. Paulus WE, Zhang M, Strehler E, El Danasouri I, Sterzik K. Influence of acupuncture on the pregnancy rate in patients who undergo assisted reproduction therapy. *Fertil Steril* 2002; 77:721–724.
201. Pei J, Sun L, Chen R, Zhu T, Qian Y, Yuan D. The effect of electro-acupuncture on motor function recovery in patients with acute cerebral infarction: a randomly controlled trial. *J Tradit Chin Med* 2001; 21:270–272.
202. Pei J, Strehler E, Noss U, Abt M, Piomboni P, Baccetti B et al. Quantitative evaluation of spermatozoa ultrastructure after acupuncture treatment for idiopathic male infertility. *Fertil Steril* 2005; 84:141–147.
203. Qin L, Gu S. Treatment of 60 Migraine Sufferers with Penetration Needling of Points and Point Selection Based on Following the Corresponding Meridians. *Int J Clin Acupunct* 2006; 15:163–167.
204. Rabl M, Ahner R, Bitschnau M, Zeisler H, Husslein P. Acupuncture for cervical ripening and induction of labor at term–a randomized controlled trial. *Wien Klin Wochenschr* 2001; 113:942–946.
205. Ramnero A, Hanson U, Kihlgren M. Acupuncture treatment during labour–a randomised controlled trial. *BJOG* 2002; 109:637–644.
206. Razavi M, Jansen GB. Effects of acupuncture and placebo TENS in addition to exercise in treatment of rotator cuff tendinitis. *Clin Rehabil* 2004; 18:872–878.
207. Reindl TK, Geilen W, Hartmann R, Wiebelitz KR, Kan G, Wilhelm I et al. Acupuncture against chemotherapy-induced nausea and vomiting in pediatric oncology Interim results of a multicenter crossover study. *Support Care Cancer* 2005; 14:172–176.
208. Ren Y, Wang D, Feng C. A comparative observation on comprehensive scalp–acupuncture treatmentof ischemic apoplectic hemiplegia. *J Tradit Chin Med* 1999; 19:200–204.
209. Resim S, Gumusalan Y, Ekerbicer HC, Sahin MA, Sahinkanat T. Effectiveness of electro-acupuncture compared to sedo-analgesics in relieving pain during shockwave lithotripsy. *Urol Res* 2005; 33:285–290.
210. Ricci L, Minardi D, Romoli M, Galosi AB, Muzzonigro G. Acupuncture reflexotherapy in the treatment of sensory urgency that persists after transurethral resection of the prostate: A preliminary report. * Neurourol Urodyn* 2004; 23:58–62.
211. Rorsman I, Johansson B. Can electroacupuncture or transcutaneous nerve stimulation influence cognitive and emotional outcome after stroke? *J Rehabil Med* 2006; 38:13–19.
212. Roschke J, Wolf C, Muller MJ, Wagner P, Mann K, Grozinger M et al. The benefit from whole body acupuncture in major depression. *J Affect Disord* 2000; 57:73–81.
213. Rosler A, Otto B, Schreiber-Dietrich D, Steinmetz H, Kessler KR. Single-Needle Acupuncture Alleviates Gag Reflex During Transesophageal Echocardiography: A Blinded, Randomized, Controlled Pilot Trial. * J Altern Complement Med* 2003; 9:847–849.
214. Rossberg E, Larsson PG, Birkeflet O, Soholt LE, Stavem K. Comparison of traditional Chinese acupuncture, minimal acupuncture at non-acupoints and conventional treatment for chronic sinusitis. *Complement Ther Med* 2005; 13:4–10.
215. Rosted P, Bundgaard M. Can acupuncture reduce the induction time of a local anaesthetic?–A pilot study. *Acupunct Med* 2003; 21:92–99.
216. Rusy LM, Hoffman GM, Weisman SJ. Electroacupuncture prophylaxis of postoperative nausea and vomiting following pediatric tonsillectomy with or without adenoidectomy. *Anesthesiology* 2002; 96:300–305.
217. Salter GC, Roman M, Bland JM, MacPherson H. Acupuncture for chronic neck pain: a pilot for a randomised controlled trial. *BMC Musculoskelet Disord* 2006; 7:99.
218. Sandberg M, Wijma K, Wyon Y, Nedstrand E, Hammar M. Effects of electro-acupuncture on psychological distress in postmenopausal women. *Complement Ther Med* 2002; 10:161–169.
219. Sangdee C, Teekachunhatean S, Sananpanich K, Sugandhavesa N, Chiewchantanakit S, Pojchamarnwiputh S et al. Electroacupuncture versus Diclofenac in symptomatic treatment of Osteoarthritis of the knee: a randomized controlled trial. *BMC Complement Altern Med* 2002; 2:3.
220. Sapir-Weise R, Berglund M, Frank A, Kristenson H. Acupuncture in alcoholism treatment: a randomized out-patient study. *Alcohol Alcohol* 1999; 34:629–635.
221. Sator-Katzenschlager SM, Wolfler MM, Kozek-Langenecker SA, Sator K, Sator PG, Li B et al. Auricular electro-acupuncture as an additional perioperative analgesic method during oocyte aspiration in IVF treatment. *Hum Reprod* 2006.
222. Scharf HP, Mansmann U, Streitberger K, Witte S, Kramer J, Maier C et al. Acupuncture and knee osteoarthritis: a three-armed randomized trial. *Ann Intern Med* 2006; 145:12–20.
223. Schmid-Schwap M, Simma-Kletschka I, Stockner A, Sengstbratl M, Gleditsch J, Kundi M et al. Oral acupuncture in the therapy of craniomandibular dysfunction syndrome – a randomized controlled trial. *Wien Klin Wochenschr* 2006; 118:36–42.
224. Schneider A, Enck P, Streitberger K, Weiland C, Bagheri S, Witte S et al. Acupuncture treatment in irritable bowel syndrome. *Gut* 2006; 55:649–654.
225. Selmer-Olsen T, Lydersen S, Morkved S. Does acupuncture used in nulliparous women reduce time from prelabour rupture of membranes at term to active phase of labour? A randomised controlled trial. *Acta Obstet Gynecol Scand* 2007; 86:1447–1452.
226. Shapira MY, Berkman N, Ben David G, Avital A, Bardach E, Breuer R. Short-term acupuncture therapy is of no benefit in patients with moderate persistent asthma. *Chest* 2002; 121:1396–1400.
227. Shen J, Wenger N, Glaspy J, Hays RD, Albert PS, Choi C et al. Electroacupuncture for control of myeloablative chemotherapy-induced emesis: A randomized controlled trial. *JAMA* 2000; 284:2755–2761.
228. Shen YF, Goddard G. The short-term effects of acupuncture on myofascial pain patients after clenching. *Pain Pract* 2007; 7:256–264.
229. Shenkman Z, Holzman RS, Kim C, Ferrari LR, DiCanzio J, Highfield ES et al. Acupressure-acupuncture antiemetic prophylaxis in children undergoing tonsillectomy. *Anesthesiology* 1999; 90:1311–1316.
230. Shlay JC, Chaloner K, Max MB, Flaws B, Reichelderfer P, Wentworth D et al. Acupuncture and amitriptyline for pain due to HIV-related peripheral neuropathy: a randomized controlled trial. Terry Beirn Community Programs for Clinical Research on AIDS. *JAMA* 1998; 280:1590–1595.
231. Si Q, Wu G, Cao X. Effects of electro-acupuncture on acute cerebral infarction. *Acupunct Electrother Res* 1998; 23:117–124.
232. Sim CK, Xu PC, Pua HL, Zhang G, Lee TL. Effects of electroacupuncture on intraoperative and postoperative analgesic requirement. *Acupunct Med* 2002; 20:56–65.
233. Skilnand E, Fossen D, Heiberg E. Acupuncture in the management of pain in labor. *Acta Obstet Gynecol Scand* 2002; 81:943–948.
234. Smith C, Crowther C, Beilby J. Acupuncture to treat nausea and vomiting in early pregnancy: a randomized controlled trial. *Birth* 2002; 29:1–9.
235. Smith C, Coyle M, Norman RJ. Influence of acupuncture stimulation on pregnancy rates for women undergoing embryo transfer. *Fertil Steril* 2006.
236. Smith MJ, Tong HC. Manual acupuncture for analgesia during electromyography: a pilot study. *Arch Phys Med Rehabil* 2005; 86:1741–1744.
237. Smith P, Mosscrop D, Davies S, Sloan P, Al-Ani Z. The efficacy of acupuncture in the treatment of temporomandibular joint myofascial pain: a randomised controlled trial. *J Dent* 2007; 35:259–267.
238. Soderberg E, Carlsson J, Stener-Victorin E. Chronic tension-type headache treated with acupuncture, physical training and relaxation training. Between-group differences. *Cephalalgia* 2006; 26:1320–1329.
239. Somri M, Vaida SJ, Sabo E, Yassain G, Gankin I, Gaitini LA. Acupuncture versus ondansetron in the prevention of postoperative vomiting. A study of children undergoing dental surgery. *Anaesthesia* 2001; 56:927–932.
240. Song Y, Zhou D, Fan J, Luo H, Halbreich U. Effects of electroacupuncture and fluoxetine on the density of GTP-binding-proteins in platelet membrane in patients with major depressive disorder. *J Affect Disord* 2007; 98:253–257.
241. Song YP, Yang W, Guo HM, Han YY. Clinical observation on acupucture combined with medicine for treatment of infantile febrile convulsion. *East West Integrat Med* 2007; 5:3–5.
242. Sprott H. Efficiency of acupuncture in patients with fibromyalgia. *Clin Bull Myofasc Pain* 1998; 3:37–43.
243. Stavem K, Kloster R, Rossberg E, Larsson PG, Dahl R, Kinge E et al. Acupuncture in intractable epilepsy: lack of effect on health-related quality of life. *Seizure* 2000; 9:422–426.
244. Stellon A, Palmer T. Acupuncture as an alternative to diazepam sedation for diagnostic gastrointestinal endoscopy. *Acupunct Med* 1999; 17:2–4.
245. Stener-Victorin E, Waldenstrom U, Nilsson L, Wikland M, Janson PO. A prospective randomized study of electro-acupuncture versus alfentanil as anaesthesia during oocyte aspiration in in-vitro fertilization. *Hum Reprod* 1999; 14:2480–2484.
246. Stener-Victorin E, Waldenstrom U, Wikland M, Nilsson L, Hagglund L, Lundeberg T. Electro-acupuncture as a peroperative analgesic method and its effects on implantation rate and neuropeptide Y concentrations in follicular fluid. *Hum Reprod* 2003; 18:1454–1460.
247. Stener-Victorin E, Kruse-Smidje C, Jung K. Comparison Between Electro-Acupuncture and Hydrotherapy, Both in Combination With Patient Education and Patient Education Alone, on the Symptomatic Treatment of Osteoarthritis of the Hip. *Clin J Pain* 2004; 20:179–185.
248. Streitberger K, Friedrich-Rust M, Bardenheuer H, Unnebrink K, Windeler J, Goldschmidt H et al. Effect of Acupuncture Compared with Placebo-Acupuncture at P6 as Additional Antiemetic Prophylaxis in High-Dose Chemotherapy and Autologous Peripheral Blood Stem Cell Transplantation: A Randomized Controlled Single-Blind Trial. *Clin Cancer Res* 2003; 9:2538–2544.
249. Streitberger K, Diefenbacher M, Bauer A, Conradi R, Bardenheuer H, Martin E et al. Acupuncture compared to placebo-acupuncture for postoperative nausea and vomiting prophylaxis: A randomised placebo-controlled patient and observer blind trial. *Anaesthesia* 2004; 59:142–149.
250. Streng A, Linde K, Hoppe A, Pfaffenrath V, Hammes M, Wagenpfeil S et al. Effectiveness and tolerability of acupuncture compared with metoprolol in migraine prophylaxis. *Headache* 2006; 46:1492–1502.
251. Sun D, Cao J. Observation of the Therapeutic Effect of Acupuncture on 58 Cases of Functional Dyspepsia. *Intl J Clin Acu* 2006; 15:169–172.
252. Sun JG, Ko CH, Wong V, Sun XR. Randomised control trial of tongue acupuncture versus sham acupuncture in improving functional outcome in cerebral palsy. *J Neurol Neurosurg Psychiatry* 2004; 75:1054–1057.
253. Sun KO, Chan KC, Lo SL, Fong DY. Acupuncture for frozen shoulder. *Hong Kong Med J* 2001; 7:381–391.
254. Sun Y, Chen H. A Controlled Study on Endometriosis Treated by Acupuncture with Shu-Mu point combination. *East West Integrat Med* 2007; 5:50–52.
255. Szczurko O, Cooley K, Busse JW, Seely D, Bernhardt B, Guyatt GH et al. Naturopathic care for chronic low back pain: a randomized trial. *PLoS ONE* 2007; 2:e919.
256. Sze FK, Wong E, Yi X, Woo J. Does acupuncture have additional value to standard poststroke motor rehabilitation? *Stroke* 2002; 33:186–194.
257. Taguchi A, Sharma N, Ali SZ, Dave B, Sessler DI, Kurz A. The effect of auricular acupuncture on anaesthesia with desflurane. *Anaesthesia* 2002; 57:1159–1163.
258. Tam LS, Leung PC, Li TK, Zhang L, Li EK. Acupuncture in the treatment of rheumatoid arthritis: a double-blind controlled pilot study. *BMC Complement Altern Med* 2007; 7:35.
259. Thomas KJ, MacPherson H, Thorpe L, Brazier J, Fitter M, Campbell MJ et al. Randomised controlled trial of a short course of traditional acupuncture compared with usual care for persistent non-specific low back pain. *BMJ* 2006; 333:623.
260. Tian X. Effect of Acupuncture and Tradtitional Chinese Herbal Medicine in Treating Endometriosis. *Intl J Clin Acu* 2006; 15:145–150.
261. Torktorabi S, Ghassemi P. Acupuncture as a palliative adjunctive Therapy in Children with Cerebral Palsy: A randomized controlled trial. *Med Acupunct* 2006; 18:42–44.
262. Trumpler F, Oez S, Stahli P, Brenner HD, Juni P. Acupuncture for alcohol withdrawal: A randomized controlled trial. *Alcohol Alcohol* 2003; 38:369–375.
263. Tsang RC, Tsang PL, Ko CY, Kong BC, Lee WY, Yip HT. Effects of acupuncture and sham acupuncture in addition to physiotherapy in patients undergoing bilateral total knee arthroplasty–a randomized controlled trial. *Clin Rehabil* 2007; 21:719–728.
264. Tseng KL, Liu HJ, Tso KY, Woung LC, Su YC, Lin JG. A Clinical Study of Acupuncture and SSP (Silver Spike Point) Electro-therapy for Dry Eye Syndrome. *Am J Chin Med* 2006; 34:197–206.
265. Tsui ML, Cheing GL. The effectiveness of electroacupuncture versus electrical heat acupuncture in the management of chronic low-back pain. *J Altern Complement Med* 2004; 10:803–809.
266. Tsukayama H, Yamashita H, Amagai H, Tanno Y. Randomised controlled trial comparing the effectiveness of electroacupuncture and TENS for low back pain: a preliminary study for a pragmatic trial. *Acupunct Med* 2002; 20:175–180.
267. Tukmachi E, Jubb R, Dempsey E, Jones P. The effect of acupuncture on the symptoms of knee osteoarthritis–an open randomised controlled study. *Acupunct Med* 2004; 22:14–22.
268. Usichenko TI, Hermsen M, Witstruck T, Hofer A, Pavlovic D, Lehmann C et al. Auricular Acupuncture for Pain Relief after Ambulatory Knee Arthroscopy–A Pilot Study. *Evid Based Complement Alternat Med* 2005; 2:185–189.
269. Usichenko TI, Dinse M, Hermsen M, Witstruck T, Pavlovic D, Lehmann C. Auricular acupuncture for pain relief after total hip arthroplasty - a randomized controlled study. *Pain* 2005; 114:320–327.
270. Vas J, Mendez C, Perea-Milla E, Vega E, Panadero MD, Leon JM et al. Acupuncture as a complementary therapy to the pharmacological treatment of osteoarthritis of the knee: randomised controlled trial. *BMJ* 2004; 329:1216–1219.
271. Vas J, Perea-Milla E, Mendez C, Navarro CS, Leon Rubio JM, Brioso M et al. Efficacy and safety of acupuncture for chronic uncomplicated neck pain: A randomised controlled study. *Pain* 2006.
272. Vickers AJ, Rees RW, Zollman CE, McCarney R, Smith CM, Ellis N et al. Acupuncture of chronic headache disorders in primary care: randomised controlled trial and economic analysis. *Health Technol Assess* 2004; 8:1–50.
273. Vickers AJ, Feinstein MB, Deng GE, Cassileth BR. Acupuncture for dyspnea in advanced cancer: a randomized, placebo-controlled pilot trial. *BMC Palliat Care* 2005; 4:5.
274. Vilholm OJ, Moller K, Jorgensen K. Effect of traditional Chinese acupuncture on severe tinnitus: a double-blind, placebo-controlled, clinical investigation with open therapeutic control. *Br J Audiol* 1998; 32:197–204.
275. Vincent A, Barton DL, Mandrekar JN, Cha SS, Zais T, Wahner-Roedler DL et al. Acupuncture for hot flashes: a randomized, sham-controlled clinical study. *Menopause* 2007; 14:45–52.
276. Waite NR, Clough JB. A single-blind, placebo-controlled trial of a simple acupuncture treatment in the cessation of smoking. *Br J Gen Pract* 1998; 48:1487–1490.
277. Wan Q. Auricular-plaster therapy plus acupuncture at zusanli for postoperative recovery of intestinal function. *J Tradit Chin Med* 2000; 20:134–135.
278. Wang B, La J, Wang BX. Clinical Studies on sciatica caused by intervertebral disc herniation with electroacupuncture or Diclofenac sodium treatment. *East West Integrat Med* 2007; 35–39.
279. Wang G, Qu F. Treatment of 482 cases of cervical spondylopathy by combining point-injection and needle-warming via moxibustion. *J Tradit Chin Med* 2001; 21:31–33.
280. Wang L. Clinical observation on acupuncture treatment in 35 cases of diabetic gastroparesis. *J Tradit Chin Med* 2004; 24:163–165.
281. Wang RR, Tronnier V. Effect of acupuncture on pain management in patients before and after lumbar disc protrusion surgery–a randomized control study. *Am J Chin Med* 2000; 28:25–33.
282. Wang SM, Peloquin C, Kain ZN. The use of auricular acupuncture to reduce preoperative anxiety. *Anesth Analg* 2001; 93:1178–80, table.
283. Wang SM, Kain ZN. Auricular acupuncture: a potential treatment for anxiety. *Anesth Analg* 2001; 92:548–553.
284. Wang SM, Maranets I, Weinberg ME, Caldwell-Andrews AA, Kain ZN. Parental auricular acupuncture as an adjunct for parental presence during induction of anesthesia. *Anesthesiology* 2004; 100:1399–1404.
285. Wang SM, Punjala M, Weiss D, Anderson K, Kain ZN. Acupuncture as An Adjunct for Sedation during Lithotripsy. *J Altern Complement Med* 2007; 13:241–246.
286. Wang Z, Li Y, Lin H. Acupuncture Treatment of Generalized Anxiety Disorder. *J TCM* 2006; 26:170-171.
287. Wayne PM, Krebs DE, Macklin EA, Schnyer R, Kaptchuk TJ, Parker SW et al. Acupuncture for upper-extremity rehabilitation in chronic stroke: a randomized sham-controlled study. *Arch Phys Med Rehabil* 2005;86:2248–2255.
288. Wedenberg K, Moen B, Norling A. A prospective randomized study comparing acupuncture with physiotherapy for low-back and pelvic pain in pregnancy. *Acta Obstet Gynecol Scand* 2000;79:331–335.
289. Weiner DK, Rudy TE, Morone N, Glick R, Kwoh CK. Efficacy of periosteal stimulation therapy for the treatment of osteoarthritis-associated chronic knee pain: an initial controlled clinical trial. *J Am Geriatr Soc* 2007; 55:1541–1547.
290. Wen D, Jiebin Y. Clinical Study on Acupuncture Treatment of Stomach Carcinoma Pain. *J Tradit Chin Med* 2002; 18:31–38.
291. Westergaard LG, Mao Q, Krogslund M, Sandrini S, Lenz S, Grinsted J. Acupuncture on the day of embryo transfer significantly improves the reproductive outcome in infertile women: a prospective, randomized trial. *Fertil Steril* 2006; 85:1341–1346.
292. White AR, Resch KL, Ernst E. Randomized trial of acupuncture for nicotine withdrawal symptoms. *Arch Intern Med* 1998; 158:2251–2255.
293. White AR, Resch KL, Chan JC, Norris CD, Modi SK, Patel JN et al. Acupuncture for episodic tension-type headache: a multicentre randomized controlled trial. *Cephalalgia* 2000; 20:632–637.
294. White P, Lewith G, Prescott P, Conway J. Acupuncture versus placebo for the treatment of chronic mechanical neck pain: a randomized, controlled trial. *Ann Intern Med* 2004; 141:911–919.
295. Widerstrom-Noga E, Dyrehag LE, Borglum-Jensen L, Aslund PG, Wenneberg B, Andersson SA. Pain threshold responses to two different modes of sensory stimulation in patients with orofacial muscular pain: psychologic considerations. *J Orofac Pain* 1998; 12:27–34.
296. Williamson L, Wyatt MR, Yein K, Melton JT. Severe knee osteoarthritis: a randomized controlled trial of acupuncture, physiotherapy (supervised exercise) and standard management for patients awaiting knee replacement. *Rheumatology (Oxford)* 2007.
297. Witt C, Brinkhaus B, Jena S, Linde K, Streng A, Wagenpfeil S et al. Acupuncture in patients with osteoarthritis of the knee: a randomised trial. *Lancet* 2005;366:136-143.
298. Witt CM, Jena S, Brinkhaus B, Liecker B, Wegscheider K, Willich SN. Acupuncture in patients with osteoarthritis of the knee or hip: a randomized, controlled trial with an additional nonrandomized arm. * Arthritis Rheum* 2006; 54:3485–3493.
299. Witt CM, Jena S, Selim D, Brinkhaus B, Reinhold T, Wruck K et al. Pragmatic Randomized Trial Evaluating the Clinical and Economic Effectiveness of Acupuncture for Chronic Low Back Pain. *Am J Epidemiol* 2006; 134:487–496.
300. Witt CM, Jena S, Brinkhaus B, Liecker B, Wegscheider K, Willich SN. Acupuncture for patients with chronic neck pain. *Pain* 2006; 125:98–106.
301. Wong AM, Leong CP, Su TY, Yu SW, Tsai WC, Chen CP. Clinical trial of acupuncture for patients with spinal cord injuries. * Am J Phys Med Rehabil* 2003; 82:21–27.
302. Wu TP, Chen FP, Liu JY, Lin MH, Hwang SJ. A randomized controlled clinical trial of auricular acupuncture in smoking cessation. *J Chin Med Assoc* 2007; 70:331–338.
303. Wyon Y, Wijma K, Nedstrand E, Hammar M. A comparison of acupuncture and oral estradiol treatment of vasomotor symptoms in postmenopausal women. *Climacteric* 2004; 7:153–164.
304. Xia Ys, Wang Jh, Shan Lj. Acupuncture plus ear-point press in preventing vomiting induced by with Cisplatin. *Int J Clin Acupunct* 2000; 11:145–148.
305. Xie H, Cao X, Huang S, Liao C. Acupuncture and Cupping for Treatment of Hiccup in Cases of Cerebrovascular Accident. *J TCM* 2006; 26:175–176.
306. Xie Q, Yang S. Clinical Observation of Combination Therapy of Acupuncture and Chinese Herbal Medicine in treating Vocal Cord Nodule. *Chin J Integrat Trad West Med* 2000; 6:56-57.
307. Xie Q, Yang S, Li W. Juvenile myasthenia laryngis treated by acupuncture and TCM medication. *J Tradit Chin Med* 2003; 23:280-281.
308. Xue CC, English R, Zhang JJ, Da Costa C, Li CG. Effect of acupuncture in the treatment of seasonal allergic rhinitis: a randomized controlled clinical trial. *Am J Chin Med* 2002; 30:1–11.
309. Xue CC, Dong L, Polus B, English RA, Zheng Z, Da Costa C et al. Electroacupuncture for tension-type headache on distal acupoints only: a randomized, controlled, crossover trial. *Headache* 2004; 44:333–341.
310. Xue CC, An X, Cheung TP, Da CC, Lenon GB, Thien FC et al. Acupuncture for persistent allergic rhinitis: a randomised, sham-controlled trial. *Med J Aust* 2007; 187:337–341.
311. Yang B, Zhang X, Song C. Clinical Observation on Acupuncture and Moxibustion Combined with Mifepristone and Misoprostol for Terminating Early Pregnancy. *Internat J Clin Acupunct* 2002; 13:263–267.
312. Yang B, Zhang C, Yang J, Chen C. Observation and Mechanism Exploration of Acupuncture on Body Weight Reduction. *J TCM* 2007; 26:250–253.
313. Yang C, Yan H. Observation of the efficacy of acupuncture and moxibustion in 62 cases of chronic colitis. *J Tradit Chin Med* 1999; 19:111–114.
314. Yeung CK, Leung MC, Chow DH. The use of electro-acupuncture in conjunction with exercise for the treatment of chronic low-back pain. *J Altern Complement Med* 2003; 9:479–490.
315. Yin C, Seo B, Park HJ, Cho M, Jung W, Choue R et al. Acupuncture, a promising adjunctive therapy for essential hypertension: a double-blind, randomized, controlled trial. *Neurol Res* 2007; 29 Suppl 1:98–103.
316. Yiu E, Xu JJ, Murry T, Wei WI, Yu M, Ma E et al. A Randomized Treatment-Placebo Study of the Effectiveness of Acupuncture for Benign Vocal Pathologies. *J Voice* 2006; 20:144–156.
317. Yu D. Clinical study of ambulatory blood pressure monitoring applied to the evaluation of acupuncture treatment for hypertension. *Chin J Integrat Trad West Med* 1998; 4:205–208.
318. Yu P, Bai H, Chen L, Zhang W, Xia Y, Wu G. Clinical study on therapeutic effect of acupuncture on Behcet's disease. *J Tradit Chin Med* 2003; 23:271–273.
319. Yu P, Zhang W, Zhao R, Wang X, Zhang S, Gao L. Study on the Correlative Law between Ultrasonic Localization and Quantitation and the Therapeutic Effect of Electroacupuncture in Cases of Urinary Calculi. *Intl J Clin Acu* 2007; 16:13–17.
320. Yurtkuran M, Kocagil T. TENS, electroacupuncture and ice massage: comparison of treatment for osteoarthritis of the knee. *Am J Acupunct* 1999; 27:133–140.
321. Zaborowska E, Brynhildsen J, Damberg S, Fredriksson M, Lindh-Astrand L, Nedstrand E et al. Effects of acupuncture, applied relaxation, estrogens and placebo on hot flushes in postmenopausal women: an analysis of two prospective, parallel, randomized studies. *Climacteric* 2007; 10:38–45.
322. Zeng X, Lei L, Lu Y, Wang Z. Treatment of heroinism with acupuncture at points of the Du Channel. *J Tradit Chin Med* 2005; 25:166–170.
323. Zhang C. The brain-resuscitation acupuncture method for treatment of post wind-stroke mental depression–a report of 45 cases. *J Tradit Chin Med* 2005; 25:243–246.
324. Zhang GJ, Shi ZY, Liu S, Gong SH, Liu JQ, Liu JS. Clinical observation on treatment of depression by electro-acupuncture combined with Paroxetine. *Chin J Integr Med* 2007; 13:228–230.
325. Zhang H, Zeng Z, Deng H. Acupuncture treatment for 157 cases of anxiety neurosis. *J Tradit Chin Med* 2003; 23:55-56.
326. Zhang J, Xu W. Frequent ventricular extrasystole treated by needling neiguan (PC 6) plus oral administration of mexiletine–a report of 30 cases. *J Tradit Chin Med* 2004; 24:40-41.
327. Zhang L. Back-front matching in treating CHD. *Int J Clin Acupunct* 2000;11:135-137.
328. Zhao J, Sang P. Clinical Observation on Acupuncture Treatment of Depressive Neurosis in 30 cases. *J TCM* 2006; 26:191-192.
329. Zhao Y. Acupuncture plus point-injection for 32 cases of obstinate urticaria. *J Tradit Chin Med* 2006; 26:22-23.
330. Zhou Y. The study of yin-yang hu ci acupuncture therapy for epilepsy. *Int J Orient Med* 2000; 25:84–87.
331. Zhu XM, Polus B. A controlled trial on acupuncture for chronic neck pain. *Am J Chin Med* 2002; 30:13–28.
332. Zhuang X, Wang L. Acupuncture treatment of Parkinson's disease–a report of 29 cases. *J Tradit Chin Med* 2000; 20:265–267.
333. Zou R, Zhang HX, Zhang TF. Comparative study on treatment of acute gouty arthritis by electroacupuncture with different frequency. *Chin J Integr Med* 2006; 12:212–214.
